# LLL12B, a small molecule STAT3 inhibitor, induces growth arrest, apoptosis, and enhances cisplatin-mediated cytotoxicity in medulloblastoma cells

**DOI:** 10.1038/s41598-021-85888-x

**Published:** 2021-03-22

**Authors:** Xiang Chen, Li Pan, Jia Wei, Ruijie Zhang, Xiaozhi Yang, Jinhua Song, Ren-Yuan Bai, Shengling Fu, Christopher R. Pierson, Jonathan L. Finlay, Chenglong Li, Jiayuh Lin

**Affiliations:** 1 Department of Biochemistry and Molecular Biology, University of Maryland School of Medicine, Baltimore, MD 21201 USA; 2 Department of Medicinal Chemistry, College of Pharmacy, The University of Florida, Gainesville, FL 32610 USA; 3 Department of Neurosurgery, Johns Hopkins University School of Medicine, Baltimore, MD 21231 USA; 4 Department of Pathology and Laboratory Medicine, Nationwide Children’s Hospital, The Department of Pathology and Department of Biomedical Education and Anatomy, The College of Medicine, The Ohio State University, Columbus, OH 43205 USA; 5 Division of Hematology, Oncology and BMT, Department of Pediatrics, College of Medicine, The Research Institute At Nationwide Children’s Hospital, The Ohio State University, Columbus, OH 43205 USA

**Keywords:** Cancer, Cancer therapy, CNS cancer

## Abstract

Signal Transducer and Activator of Transcription 3 (STAT3) is a transcription factor and an oncogene product, which plays a pivotal role in tumor progression. Therefore, targeting persistent STAT3 signaling directly is an attractive anticancer strategy. The aim of this study is to test the efficacy of a novel STAT3 small molecule inhibitor, LLL12B, in suppressing medulloblastoma cells in vitro and tumor growth in vivo. LLL12B selectively inhibited the induction of STAT3 phosphorylation by interleukin-6 but not induction of STAT1 phosphorylation by INF-γ. LLL12B also induced apoptosis in human medulloblastoma cells. In addition, LLL12B exhibited good oral bioavailability in vivo and potent suppressive activity in tumor growth of medulloblastoma cells in vivo. Besides, combining LLL12B with cisplatin showed greater inhibition of cell viability and tumorsphere formation as well as induction of apoptosis comparing to single agent treatment in medulloblastoma cells. Furthermore, LLL12B and cisplatin combination exhibited greater suppression of medulloblastoma tumor growth than monotherapy in vivo. The present study supported that LLL12B is a novel therapeutic agent for medulloblastoma and the combination of LLL12B with a chemotherapeutic agent cisplatin may be an effective approach for medulloblastoma therapy.

## Introduction

Medulloblastoma is one of the most common malignant central nervous system tumor in childhood^1^. Although the 5-year survival rate of 80% is achieved in average-risk patients, the prognosis of high-risk patients remains poor^2^. Conventional surgery/radiation therapy followed by adjuvant chemotherapy is effective in shrinking primary medulloblastoma^3,4^ and many different chemotherapeutic regimens have been developed after radiotherapy. Encouraging results have been found with the use of vincristine during radiotherapy followed by cycles of lomustine (CCNU), cisplatin, and vincristine^5^. Unfortunately, children survived medulloblastoma suffer from severe long-term neurologic, cognitive, and endocrinologic sequelae due to cranial irradiation and high-dose chemotherapy^6^. Currently, a major challenge for improving medulloblastoma therapy is the development of novel treatment agents, especially oncogene-targeted approaches that show more effective and less long-term neurotoxic side effects.

Of the seven members of the signal transducer and activator of transcription family proteins (STAT 1, 2, 3, 4, 5a, 5b, and 6), STAT3 is known as both a transcription factor and an oncogene, and is considered to be one of the most important regulators for cancer progression^7^. Constitutively activated STAT3 is strongly associated with a variety of human solid tumors and hematologic malignancies, including brain tumors^8,9^. Abundant evidence suggests that STAT3 contributes to cancer cell proliferation, survival, tumor angiogenesis, invasion, migration and resistance to apoptosis, since the activated STAT3 complex translocates to the nucleus to regulate STAT3 target gene expression that is involved in the cell cycle (Cyclin D1 and c-Myc), survival (Survivin, Bcl-xL and Bcl-2), angiogenesis (VEGF), and invasion/migration (MMP-2)^10–12^. Phosphorylated STAT3 also promotes cancer stem cells (CSCs) self-renewal and differentiation^13^.

Given its pivotal role in both tumor onset and progression, STAT3 signaling has emerged as an attractive target for small molecule therapeutics. Currently, multiple small-molecule inhibitors targeting STAT3 signaling are in various phases of clinical development^14–19^. Indirect inhibitors block upstream effectors of STAT3, such as cytokines and kinases. JAK is an upstream kinase of STAT3. JAK inhibitors, such as ruxolitinib and tofacitinib, have been approved by the FDA for the treatment of myelofibrosis and rheumatoid arthritis respectively^15^. WP-1066 and AZD1480 are being tested in clinical trials. However, JAK inhibitors have limitations that observed in clinical trials, including off-target neurotoxicity and rate-limiting toxicities^20^. Furthermore, targeting one of the upstream effectors of STAT3 is unlikely to be sufficient for cancer therapy, because multiple upstream activators converge on the STAT3 signaling. Direct inhibitors target the SH2, DNA-binding, or N-terminal domains of STAT3. Peptides and peptidominetics inhibitors could disrupt the dimerization of STAT3 and effectively blocks its transcriptional activity, but these inhibitors show low cell permeability and stability. STAT3 targeting decoy oligonucleotides and small interfering RNA inhibitors seem like a viable means to selectively inhibit STAT3 activity, yet they have low cellular delivery and relative instability^21,22^.

With advances in medicinal chemistry, structure-based drug design is becoming a robust tool for drug development^23^. In our previous studies, we established a novel approach termed advanced ligand simultaneous docking (AMLSD) and developed several new small molecule inhibitors targeting STAT3^24–29^. In the present study, we identified a novel small molecular STAT3 inhibitor, LLL12B, and demonstrated that LLL12B inhibits STAT3 phosphorylation and induces apoptosis in medulloblastoma cells in vitro and suppresses tumor growth in vivo. Importantly, the present study provided a new rationale for combinational therapies, in which anti-STAT3 small molecule inhibitor is synergistically cooperated with current regimen, cisplatin, in medulloblastoma. Our findings support the potential of testing novel small molecule inhibitor LLL12B in combination with cisplatin for the treatments of medulloblastoma.

## Methods

### Reagents and antibodies

Cisplatin was purchased from Sigma and was dissolved in ddH_2_O at a stock concentration of 5 mM in the dark. LLL12B was synthesized at the University of Florida College of Pharmacy. LLL12B powder was dissolved in sterile dimethyl sulfoxide (DMSO) to make a 20 mM stock solution and stored at − 20 °C. 3-(4, 5-dimethylthiazol-2-yl)-2, 5-diphenyltetrazolium bromide (MTT) was purchased from Sigma, and N, N-dimethylformamide (DMF) was obtained from Fisher. IL-6 and Interferon (INF-γ) were purchased from Cell Signaling. The primary antibodies, phosphorylated STAT3 (Y705), STAT3, P-ERK, ERK, P-AKT, AKT, phosphorylated STAT1 (Y701), STAT1, CyclinD1, Survivin, and GAPDH as well as the horseradish peroxidase-labeled anti-rabbit secondary antibodies were obtained from Cell Signaling.

### Cell culture

Human medulloblastoma cell lines D425, UW426 and UW288 were kindly provided by Dr. Hui-Wen Lo (Wake Forest School of Medicine) and Dr. Corey Raffel (University of California San Francisco). Medulloblastoma cell line D283 and DaoY was purchased from American Type Culture Collection (ATCC). Cells were maintained in Dulbecco’s modified Eagle’s medium (DMEM, Mediatech, #10013 CV) with L-glutamine supplemented with 10% fetal bovine serum (FBS) and 1% penicillin/streptomycin. All cells were cultured in a humidified 37 °C incubator with 5% CO_2_. Tumorspheres of D425 and D283 were cultured in PromoCell 3D tumorsphere medium XF supplemented with SupplementMix (PromoCell, #C-28070).

### Computational docking model

LLL12B was docked to the SH2 domain of STAT3 using AutoDock4^30^ with LGA (Lamarckian Genetic Algorithm) by Dr. Chenglong Li’s laboratory. LLL12B chemical structure was also provided by Dr. Chenglong Li’s laboratory. The protein and ligand were protonated and Gasteiger charged. AutoGrid affinity map was computed for all atom types. The population was set at 100 and 10 million energy evaluations were completed. With RMSD (root mean square deviation of 1.5 Å, the major lowest energy cluster was identified with 63% conformers and binding energy of -7.9 kcal/mol. The image was created with graphic software called PMV in MGLTools, version 1.5.6 (URL: http://mgltools.scripps.edu/).

### Apoptosis assay

Cells were plated in 96-well plates (8000–12,000 cells/well) and allowed to adhere overnight. Cells were treated with LLL12B or DMSO only. After 5 h treatment, active caspase-3/7 were measured using Caspase-3/7 Fluorescence Assay Kit (Cayman, Ann Arbor, MI) according to the manufacture’s instruction. Briefly, cells were centrifuged in the plate (800 *g*, 5 min) and the culture medium was aspirated, and then 200 µL of assay buffer was added to each well to wash the cells and the supernatant was removed after centrifuge. After that, 100 µL of cell-based assay lysis buffer was added to each well with shaking for 30 min at room temperature. Subsequently, 90 µL of the supernatant from each well was transferred to a corresponding well in a new black 96-well plate and 10 µL of assay buffer was added to appropriate wells. The plate was incubated at 37 °C for 90 min followed by addition of 100 µL of the caspase-3/7 substrate solution. Finally, the fluorescence intensity of each well was read at excitation 485 nm and emission 535 nm. All treatments were performed in triplicate.

### Western blot analysis

Cells was harvested and extracted using cell lysis buffer (Cell Signaling) supplemented with phosphatase and protease inhibitors. Tumors from mice were grinded in the liquid nitrogen, and extracted proteins using cell lysis buffer supplemented with phosphatase and protease inhibitors. Extracted protein samples were separated by 10% SDS-PAGE and transferred to polyvinylidene difluoride membranes. After blocking with TBS-T buffer containing 5% skim milk (Lab Scientific Inc) at room temperature for 1 h, each membrane was incubated at 4 °C overnight with one of the following primary antibodies at 1:1,000 dilution: rabbit anti-phosphorylated STAT3 (Y705), rabbit anti-STAT3, rabbit anti-phosphorylated STAT1 (Y701), rabbit anti-STAT1, rabbit anti-Cyclin D1, rabbit anti-Survivin, and rabbit anti-GAPDH. Subsequently, each membrane was washed with TBS-T and incubated with horseradish peroxidase-conjugated secondary antibody (1:10,000) at room temperature for 1.5 h. Proteins of interest were visualized using SUPERSIGNAL West Femto Maximum Sensitivity Substrate (Thermo, #34096). Quantification was performed using ImageJ.

### STAT3 siRNA transfection and viability assay

SignalSilence STAT3 siRNA I was purchased from Cell Signaling (#6580) and medulloblastoma cells were transfected using Lipofectamine 2000 reagent (Invitrogen). The sequence of STAT3 siRNA: GGUCCCUCAUCCUGUUUGU, ACAAACAGGAUGAGGGACC. AMBION SILENCER Negative Control siRNA (#AM4611) was purchased from Invitrogen. Cells were seeded in 96-well plates (8,000 cells/well) and allowed to adhere overnight. siRNA and Lipofectamine 2000 were diluted at 1:50 in Opti-MEM medium respectively. Gently mixed and incubated at room temperature for 5 min, and then the diluted siRNA was added to the diluted Lipofectamine 2000 reagent (1:1 ratio) with an additional incubation of 25 min. The final concentration of siRNA was 100 nM. After that, culture medium was replaced by Lipofectamine 2000 reagent: siRNA complexes and cultured for 24 h. After transfection, cells were treated with LLL12B or DMSO only for 48 h, and then cell viability was measured by MTT assay.

### MTT assay

Cells were seeded in 96-well plates at a density of 3,000 cells. After overnight attachment, cells were treated with different concentrations of LLL12B and/or cisplatin or DMSO alone. After 72 h treatment, 20 μL of MTT solution was added for 4 h of additional culture, and then 150 μL of DMF solubilization solution was added with shaking overnight in the dark. Absorbance was measured at 595 nm. Combination index (CI) was calculated using ComuSyn software^31^. The value of CI < 1 represents a synergistic effect, > 1 means an antagonistic effect, and equal to 1 indicates an additive effect.

### Tumorsphere formation

Cells were seeded (10,000 cells/mL) in ultra-low attachment 6-wells plates and treated with different concentrations of LLL12B and/or cisplatin or DMSO alone for 8 days. One-half of the culture volume of fresh 3D Tumorsphere Medium XF was added every 4 days. Images were captured on a Nikon Eclipse TS100 microscope. Original magnification, × 10.

### Mouse studies in vivo

All animal studies were carried out in accordance with the standard procedures approved by the Institutional Animal Care and Use Committee (IACUC) of University of Maryland School of Medicine and University of Florida respectively. Furthermore, our animal studies were performed in compliance with the ARRIVE guidelines. Cells (1 × 10^7^ cells/sites) were suspended in PBS mixed with matrigel (1:1, Corning, # 354234) and injected subcutaneously into the flank region of 4 to 5 week-old female athymic nude mice (Envigo). When the volume of the tumors reached approximately 100 mm^3^, mice were randomized into four groups: (1) LLL12B (2.5 mg/kg) treated orally once daily only, (2) cisplatin (5 mg/kg) intraperitoneal injection treated once every four days only, (3) combination of LLL12B (2.5 mg/kg) oral once daily and cisplatin (5 mg/kg) intraperitoneal injection once every four days and (4) vehicle only once daily. Tumor growth was determined by measuring the minor (*W*) and major (*L*) diameter once every other day with a caliper, and calculated according to the following equation: tumor volume = 0.5236 × *L* × *W*^2^. Body weight and clinical signs were monitored once every other day. In vivo PK studies in mice were performed by Dr. Chenglong Li’s laboratory at the University of Florida.

### Statistical analysis

All data are presented as mean ± standard error (SE). The difference between two groups was analyzed by Student’s *t*-test or ANOVA as appropriate. All statistical calculations were performed on the GraphPad software. (Graphpad Software, Inc.’; *P < 0.05, **P < 0.01, and ***P < 0.001).

## Results

### LLL12B inhibits STAT3 phosphorylation and reduces downstream target protein expression in medulloblastoma cells

**Figure 1:**
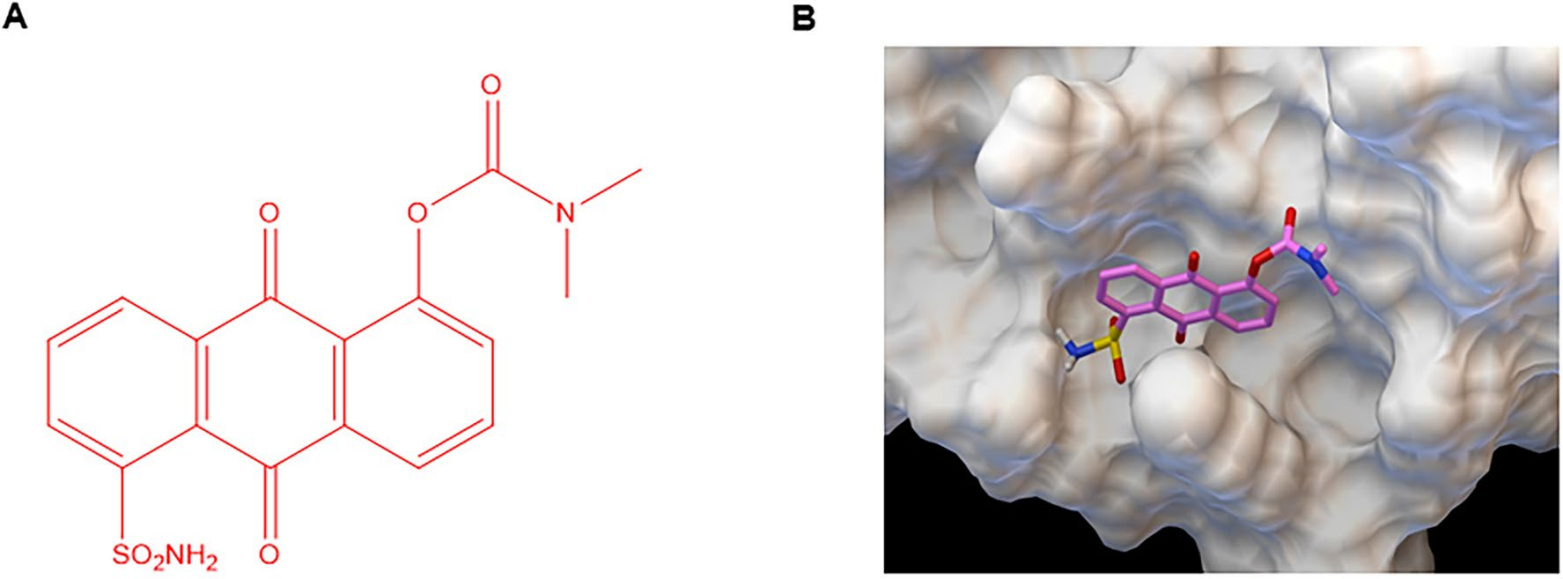
(**A**) The chemical structure of LLL12B. (**B**) A docking model of LLL12B (ball-and-stick) binding to the SH2 domain of STAT3 (surface representation). The image was created with graphic software called PMV in MGLTools, version 1.5.6 (URL: http://mgltools.scripps.edu/).

A computational-based drug design approach, AMLSD, was applied to identify the fragments from known STAT3 inhibitors that target the STAT3 Src Homology 2 (SH2) domain^29^. A non-peptide small molecular compound, LLL12B (Fig. 1A), which has low molecular weight and high potency and selectivity through binding to SH2 domain dimerization interface was selected. LLL12B docking to SH2 domain yields a major cluster with binding energy of − 7.9 kcal/mol, converted to binding Kd of 1.6 µM. Figure 1B showed its tight binding to the SH2 domain pTyr705 binding pocket with surface representation. The major interactions are the two hydrogen bonds between Arg609 and the quinone carbonyl oxygen and sulfonyl oxygen, respectively; the Lys591 cation—π electrons of LLL12B and the hydrogen bonding between carbamate carbonyl and amide of Ser636. In addition, we performed new in vivo pharmacokinetics (PK) studies of LLL12B and LLL12 orally (PO) and showed LLL12B exhibited extensive in vivo bioavailability comparing to its original compound LLL12, as shown in Table 1. When one time of 8 mg/kg of LLL12B and 20 mg/kg of LLL12 respectively were given to mice, the oral T_max_ of LLL12B increases to almost 8 h from 5 min of LLL12 and C_max_ is more than doubled in LLL12B compared to LLL12. Importantly, the oral bioavailability (F) of LLL12B increased to 47.7% from 2.73% of LLL12 (Table 1). Because LLL12B showed superior in vivo PK and oral bioavailability than its parent STAT3 inhibitor LLL12, we next tested its biological activity to inhibit medulloblastoma cells in vitro and suppress medulloblastoma tumor growth in vivo.

**Table 1:** Relative bioavailability of LLL12 and LLL12B in mice dosed PO.

PK parameters	Unit ↓ (Dosage →)	LLL12	LLL12B
		PO	PO
		20 mg/kg	8 mg/kg
T_max_	hours	0.083	7.68
C_max_	nM	176	278
AUC_all_	nM*hr	40.7	1357
Bioavailability (F)	%	2.73	47.7

per os (PO): by mouth or orally.

**Figure 2:**
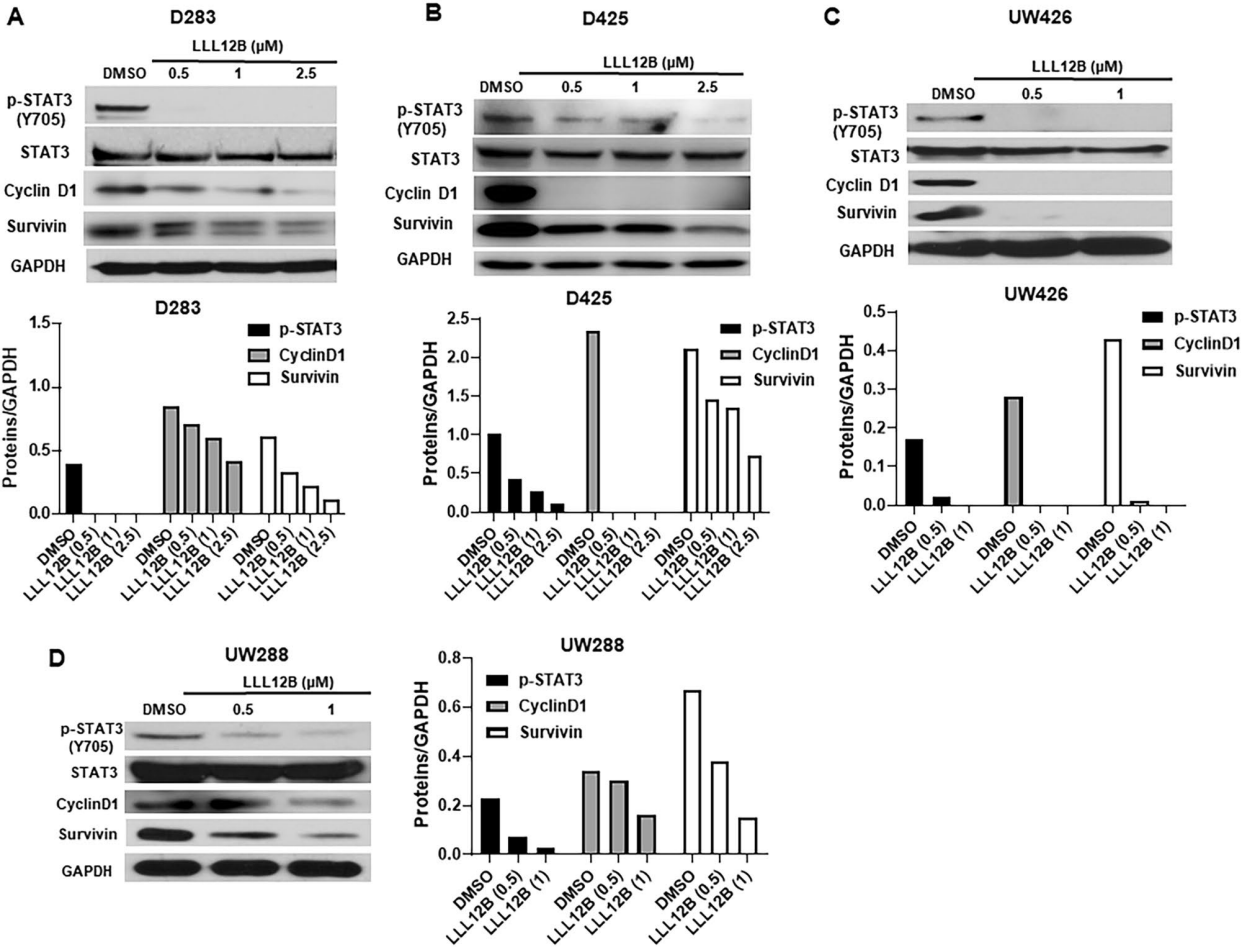
LLL12B inhibits STAT3 phosphorylation and the expression of its downstream targets, CyclinD1 and Survivin in D283 (**A**), D425 (**B**), UW426 (**C**), and UW288 (**D**) human medulloblastoma cells. Cells were seeded and allowed to adhere overnight, and then treated with different concentrations of LLL12B (from 0.5 to 2.5 μM) for 16 h. P-STAT3 (Y705), STAT3, CyclinD1, Survivin and GAPDH levels were analyzed by Western blots.

**Figure 3:**
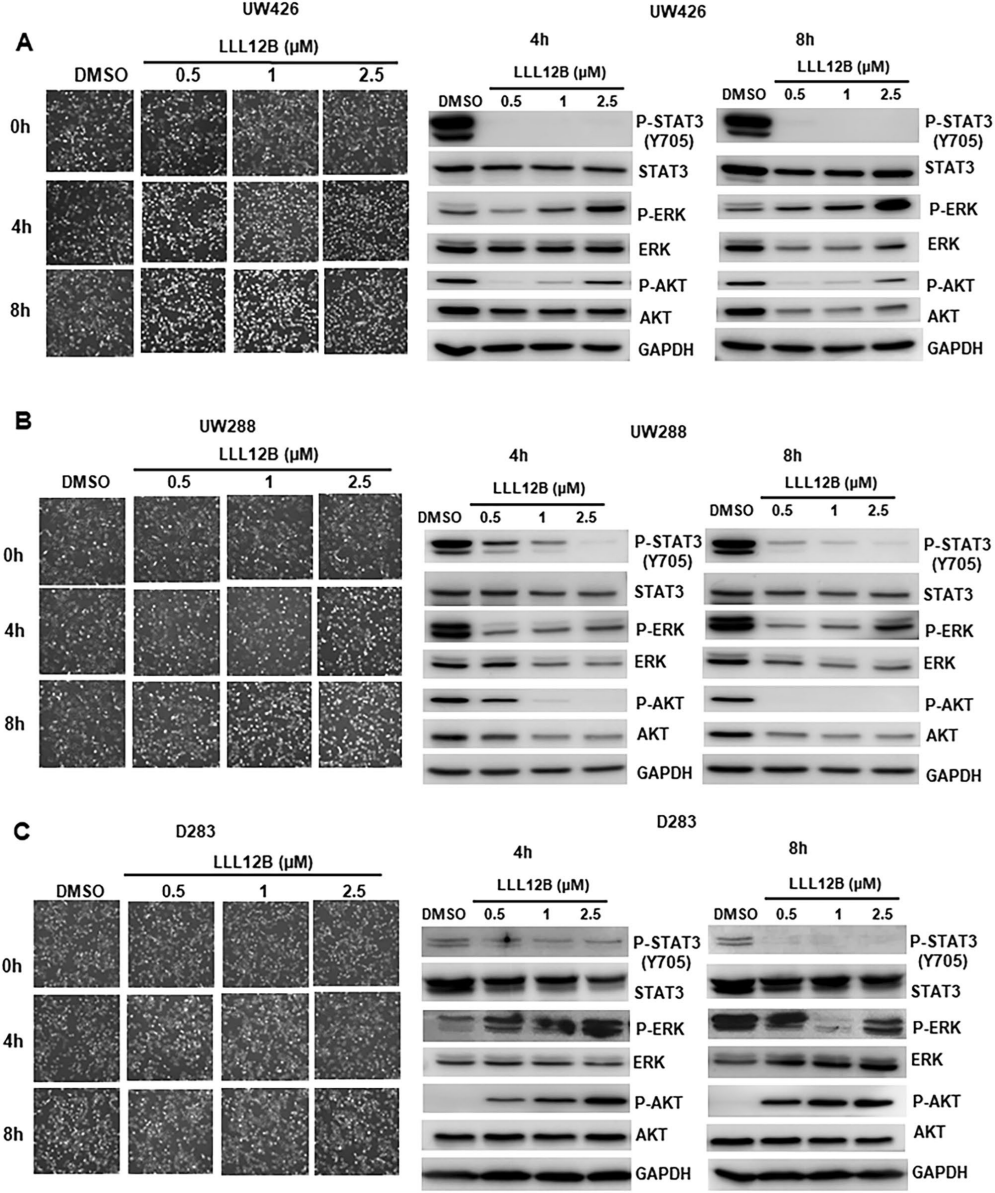
Images of cells treated with LLL12B and Western blot analysis of P-STAT3 (Y705), STAT3, P-ERK, ERK, P-AKT, AKT, and GAPDH after treatment with LLL12B in UW426 (**A**), UW288 (**B**), and D283 (**C**) human medulloblastoma cells. Cells were seeded and allowed to adhere overnight, and then treated with different concentrations of LLL12B (from 0.5 to 2.5 μM) for 4 or 8 h.

To evaluate the potency of LLL12B, D283, D425, UW426 and UW288 medulloblastoma cells were treated with different concentrations of LLL12B (from 0.5 to 2.5 μM) for 16 h. As a result, LLL12B dose-dependently suppressed STAT3 phosphorylation (Y705) (Fig. 2). Given that phosphorylated STAT3 could initiate transcription of target genes that are associated with tumor cell growth and apoptosis, the expression levels of STAT3 downstream targets were determined. The results showed that both cell cycle gene Cyclin D1 and anti-apoptosis gene Survivin were downregulated after LL12B treatments (Fig. 2). To exclude the possibility that cell density may affect a role to affect P-STAT3, we performed experiments using UW426, UW288, and D283 medulloblastoma cell lines and seeded the same density of cells with LLL12B using shorter time points 4 and 8 h. Our results showed that the cell density in all cell lines treated with LLL12B, 0.5, 1.0, and 2.5 μM are very similar to DMSO-no drug group (Fig. 3). P-STAT3 levels were significantly reduced by LLL12B at 4 and 8 h time points in these medulloblastoma cell lines (Fig. 3). We examined P-ERK or P-AKT as controls and found that LLL12B 0.5 and 1.0 μM reduced P-AKT but P-AKT at LLL12B 2.5 μM bound back in UW426 cells. LLL12B induced P-ERK in UW426 cells (Fig. 3A). In UW288 cells, LLL12B has some inhibition of both P-AKT and P-ERK (Fig. 3B). In D283 cells, LLL12B induced P-AKT and did not generally inhibit P-ERK (Fig. 3C).

### LLL12B inhibits IL-6 stimulated STAT3 but not INF-γ induced STAT1 in medulloblastoma cells

**Figure 4:**
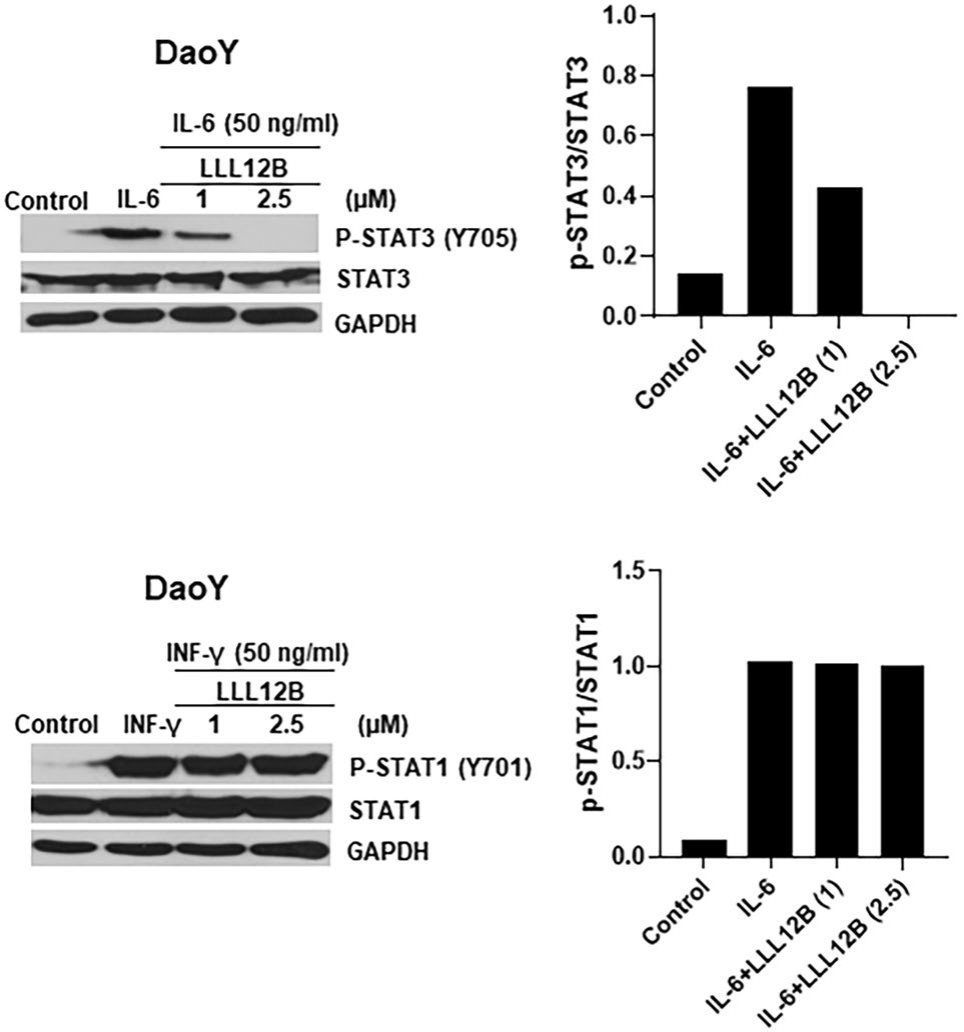
Inhibitory effect of LLL12B on IL-6 stimulated P-STAT3 and INF-γ induced P-STAT1 in DaoY medulloblastoma cells. DaoY cells were seeded and allowed to adhere overnight, and then the medium was changed to DMEM with 0% FBS. Cells were treated with LLL12B or DMSO for 4 h, and then 50 ng/ml of IL-6 or INF-γ was added for 30 min and cells were harvested for Western blot. For the control group, cells were treated only with DMSO and neither IL-6 nor INF-γ was added.

To verify the selective toxicity of LLL12B, DaoY human medulloblastoma cell line was used and treated with LLL12B (1 and 2.5 μM) or DMSO for 4 h followed by induction of IL-6 or INF-γ (50 ng/mL) for 30 min. As shown in Fig. 4, IL-6 alone significantly induced STAT3 phosphorylation and INF-γ alone notably induced STAT1 phosphorylation. Importantly, LLL12B significantly inhibited IL-6 induced phosphorylation of STAT3 but not INF-γ stimulated STAT1 phosphorylation. These results indicated that the LLL12B selectively inhibited STAT3 phosphorylation without affecting the induction of phosphorylated STAT1 protein by INF-γ.

### LLL12B suppresses cell viability through targeting STAT3 in medulloblastoma cells

**Figure 5:**
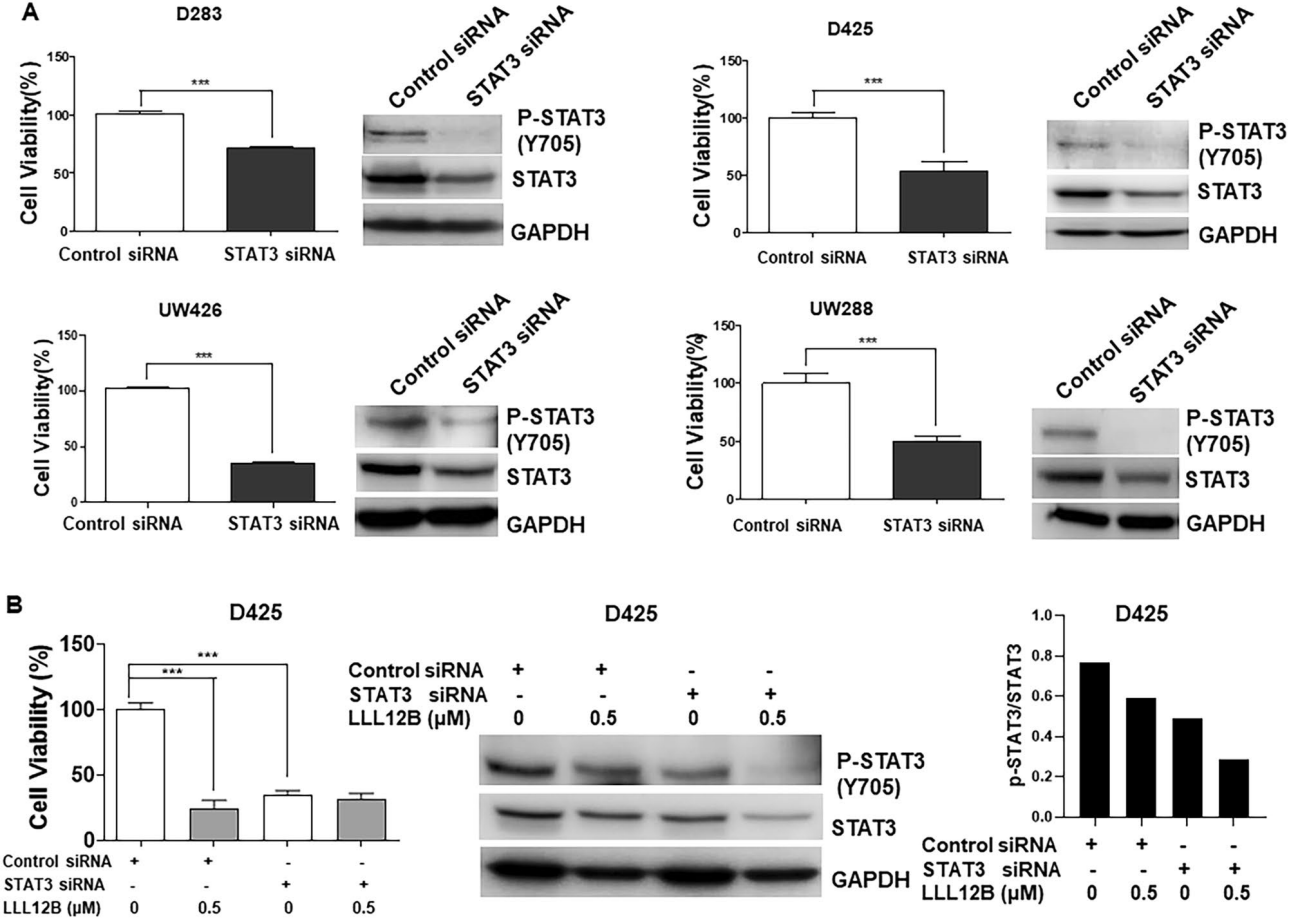
(**A**) Knock down of STAT3 by STAT3-specific siRNA inhibits STAT3 phosphorylation, STAT3 protein level and cell viability in D283, D425, UW426, and UW288 human medulloblastoma cell lines compared to Control siRNA. (**B**) Inhibition of STAT3 phosphorylation and cell viability by LLL12B or STAT3 siRNA. Cells were seeded for overnight, and transiently transfected with 100 nM of STAT3 siRNA or control siRNA for 24 h. After that, cells were cultured for 48 h then were treated with LLL12B (0.5 μM) or DMSO for additional 48 h. Cell viability was measured using MTT assay. Experiments were performed in triplicate and data are presented as means ± SE. ***P < 0.001.

To further elucidate the mechanism of LLL12B in medulloblastoma cells, firstly, STAT3 was knockdown in D283, D425, UW426 and UW288 cells using STAT3 siRNA. Control siRNA was used as control. To confirm the knockdown efficiency, western blot was performed after transfection and the result indicated that STAT3 was successfully knockdown compared to control (Fig. 5A). In addition, the cell viability of these cell lines significantly decreased after STAT3 knockdown compared to the control, which suggested that STAT3 plays an important role in promoting cancer cell growth. Next, to investigate whether STAT3 siRNA and LLL12B inhibit the same STAT3 pathway, D425 cells were treated with LLL12B (0.5 μM) combined with STAT3 knockdown. As a result, LLL12B could impede cell viability yet no synergistic effect was observed when combined treatment of LLL12B and STAT3 siRNA were used, which support that STAT3 is a target of LLL12B and is required for LLL12B’s activity to suppress the cell viability (Fig. 5B). In addition, STAT3 siRNA and LLL12B combination seemed to further inhibit the STAT3 protein and P-STAT3 level but not cell viability, suggesting that cell viability of D425 medulloblastoma cells are not only depend on STAT3 pathway alone (Fig. 5B).

### LLL12B treatment significantly suppressed tumor growth of medulloblastoma in vivo

**Figure 6:**
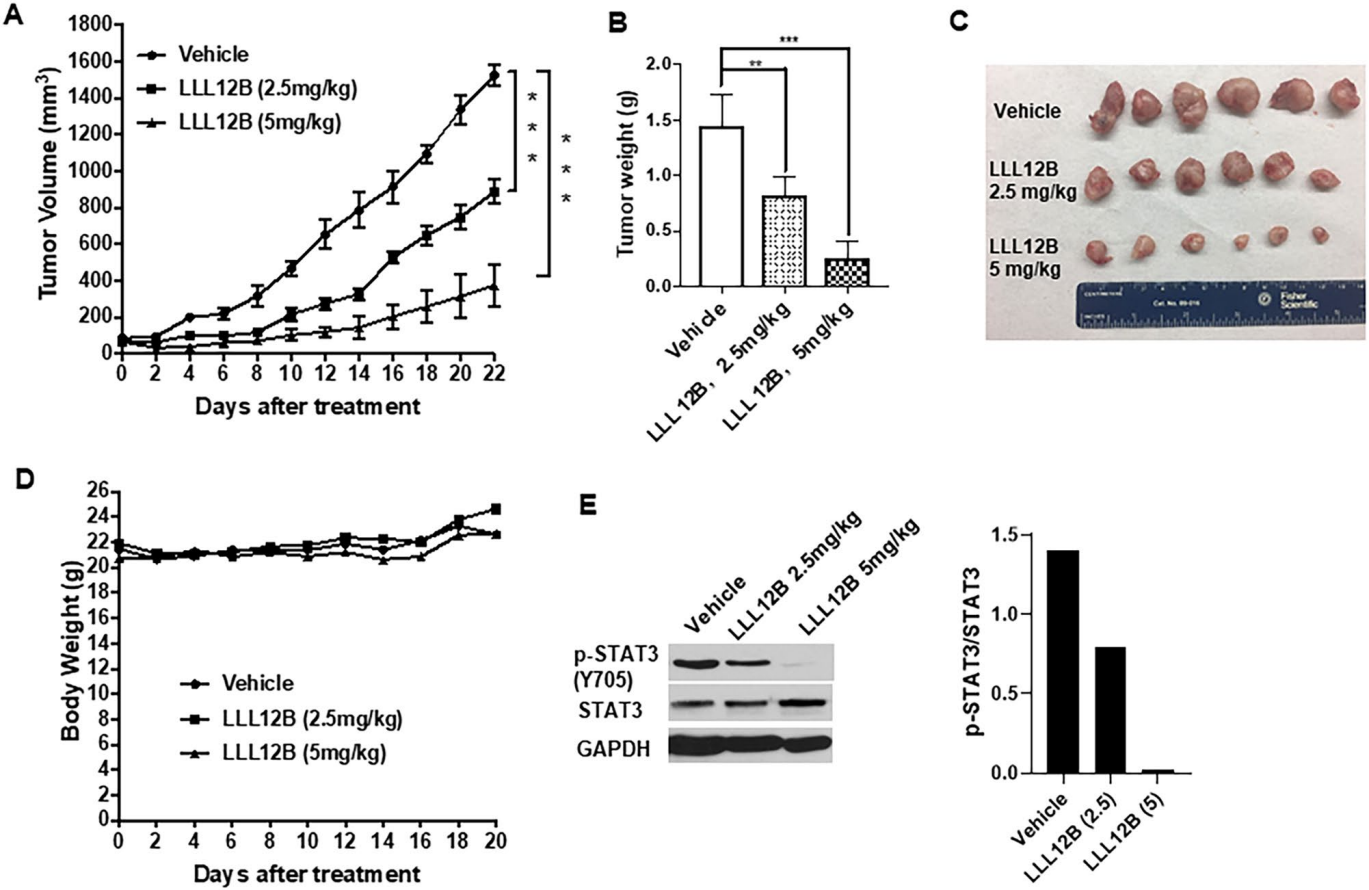
LLL12B suppressed tumor growth in medulloblastoma xenografts. (**A**) Tumor volume, (**B**) Tumor weights. (**C**) Tumor samples. (**D**) Body weights. (**E**) Western blot of tumor samples. The protein expression levels of P-STAT3(Y705), STAT3, and GAPDH. D283 cells (1 × 10^7^ cells/tumor) were harvested and suspended in PBS and then implanted subcutaneously with matrigel (1:1 ratio) into the flank region of 4 to 5 week-old female athymic nude mice. Mice were randomized into three groups (6 mice for each group) and treated with LLL12B (2.5 mg/kg or 5 mg/kg, oral gavage, once daily).

Based on the in vitro results, we moved on to in vivo and assessed the therapeutic effects of LLL12B in D283 tumor xenograft model. As presented in Fig. 6A–C, administration of LLL12B suppressed tumor growth and tumor weights by ~ 40% and ~ 75% at 2.5 mg/kg and 5 mg/kg, respectively, compared to their vehicle-treated control. Notably, LLL12B has no significant impact on the body weight of mice (Fig. 6D), suggesting the low toxicity on mice. Furthermore, the expression level of P-STAT3(Y705) directly from tumors was determined and the result showed that P-STAT3(Y705) level was dose-dependently down-regulated after LLL12B treatment comparted to the vehicle group (Fig. 6E).

### LLL12B and cisplatin combination exhibited potent activity in inhibiting medulloblastoma cell viability

**Figure 7:**
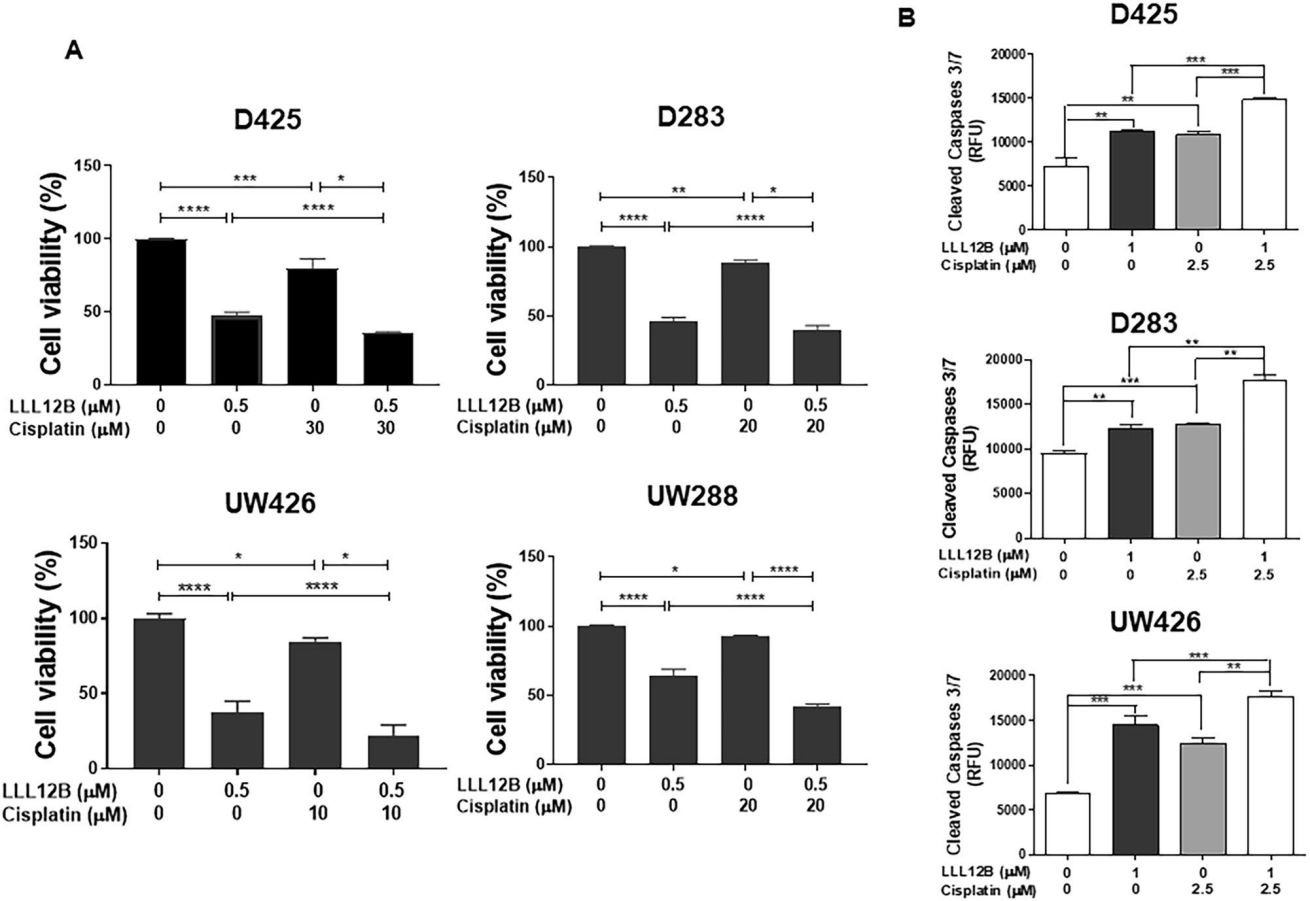
(**A**) Inhibitory effect of LLL12B and cisplatin combination synergistic inhibit the cell viability in D283, D425, UW426 and UW288 human medulloblastoma cells. Cells (3000 cells/well) were seeded in 96-well plates for overnight, cells were treated with LLL12B (0.5 μM), cisplatin (10, 20, or 30 μM), drug combination, or vehicle alone for 72 h. After treatment, cell viability was determined using the MTT assay. (**B**) Apoptotic induction of the combination of LLL12B and cisplatin in D283, D425, and UW426 medulloblastoma cells. Cells were seeded in 96-well plates (12,000 cells/well) for overnight. Cells were treated with LLL12B, cisplatin, drug combination or vehicle for 5 h, then active caspase-3/7 were measured using the Caspase-3/7 Fluorescence Assay Kit. Data are presented as means ± SE. Experiments were performed in triplicate and data are presented as means ± SE. *P < 0.05, **P < 0.01, and ***P < 0.001.

Cisplatin is an adjuvant chemotherapeutic agent used in the treatment of medulloblastoma, and chemo-resistance has been a major clinical impediment. To investigate whether LLL12B has a synergistic effect when combined with cisplatin, a series of concentrations of LLL12B and cisplatin were first tested in D283, D425, UW426 and UW288 cells and the suitable concentrations for combination were determined on the cell viability. Next, two concentrations of LLL12B and cisplatin were selected in the combinational treatment. As a result, the combination of LLL12B (0.5 μM) and cisplatin showed more significantly inhibition on the cell viability than the single drug alone (Fig. 7A). Moreover, the CI values in the combination of LLL12B and cisplatin showed 0.20, 0.65, 0.67, and 0.61 in D425, D283, UW426 and UW288 cells, respectively (Fig. 7A). CI < 1 supporting that LLL12B and cisplatin combination have synergistic inhibitory effects in D283, D425, UW426 and UW288 human medulloblastoma cells.

### LLL12B and cisplatin combination induced significant apoptosis in medulloblastoma cells

To further evaluate the effect of combining LLL12B with cisplatin, apoptosis was tested through the measure of active or cleaved caspase-3/7. D425, D283, and UW426 medulloblastoma cells were treated with LLL12B (1 μM) and cisplatin (2.5 μM) or single drug alone. As shown in Fig. 7B, LLL12B or cisplatin alone increased caspase-3/7 activation. Moreover, the induction of apoptosis was more significantly induced compared to that of single agent treatment alone. This result suggested that combinational treatment of LLL12B and cisplatin is more effective at inducing medulloblastoma cell death than either drug when it is used alone.

### LLL12B inhibits tumorsphere formation and generates synergy with cisplatin in medulloblastoma cells

**Figure 8:**
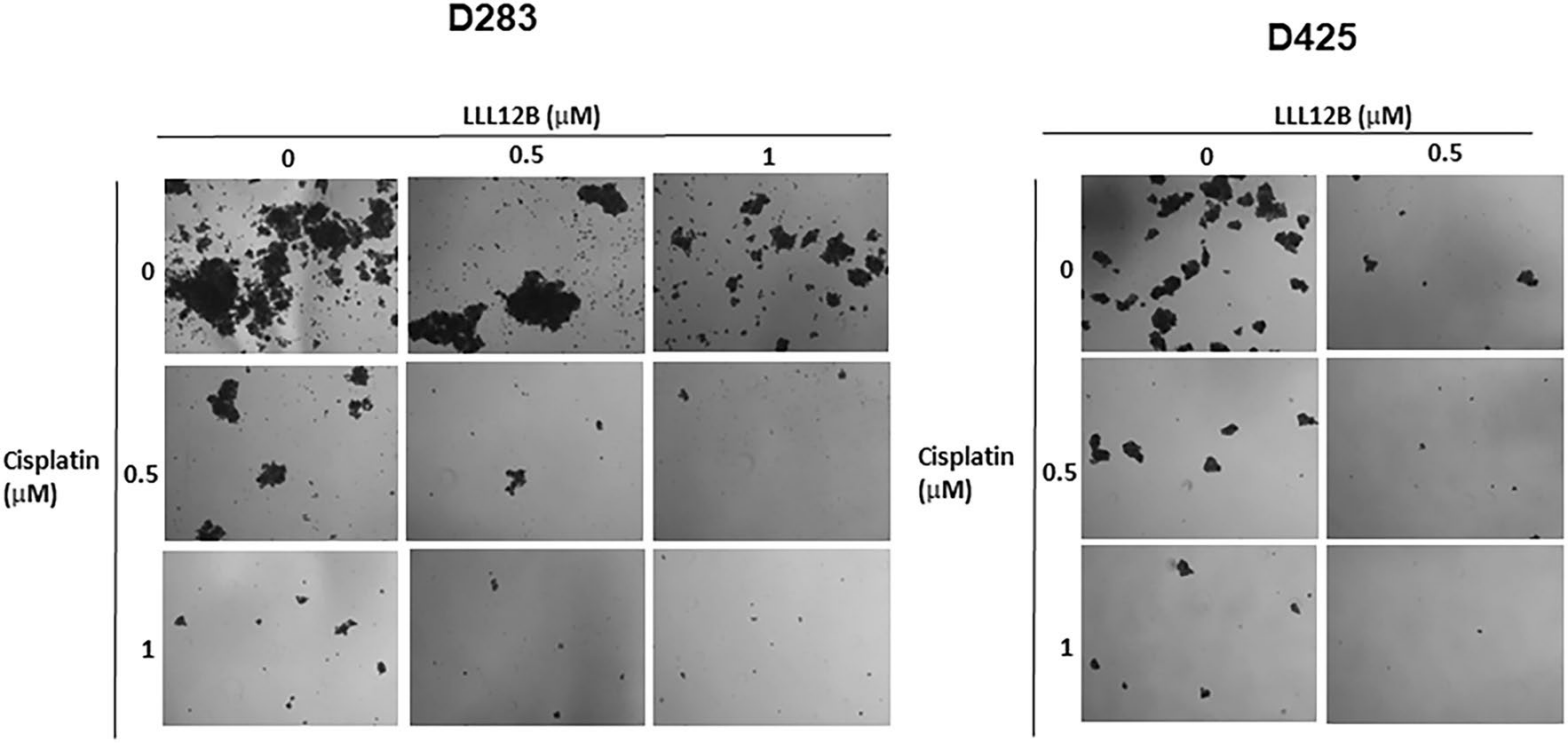
Inhibitory effect of tumorsphere formation after combination treatment with LLL12B and cisplatin. Cells were plated in ultra-low attachment 6-wells at 10,000 cells/mL and treated with LLL12B (0.5 and 1 μM) and/or cisplatin (0.5 and 1 μM) for 8 days. Images were captured on a Nikon Eclipse TS100 microscope. Original magnification, × 10.

Next, the effects of LLL12B on tumorsphere formation was tested using D283 and D425 medulloblastoma cells. Both D283 and D425 cells could grow as non-adherent and anchorage-independent and formed varied numbers and sizes of tumorspheres. However, after the treatment with LLL12B (0.5 and 1 μM), numbers and sizes of tumorspheres decreased with accumulation of smaller tumorspheres which eventually disintegrated (Fig. 8). Furthermore, D283 and D425 cells were treated with the combination of LLL12B (0.5 and 1 μM) and cisplatin (0.5 and 1 μM). The result indicated that the combination of LLL12B and cisplatin further inhibit the formation of tumorspheres D283 and D425 medulloblastoma cells (Fig. 8).

### LLL12B acts in synergy with cisplatin on suppressing medulloblastoma tumor growth in vivo

**Figure 9:**
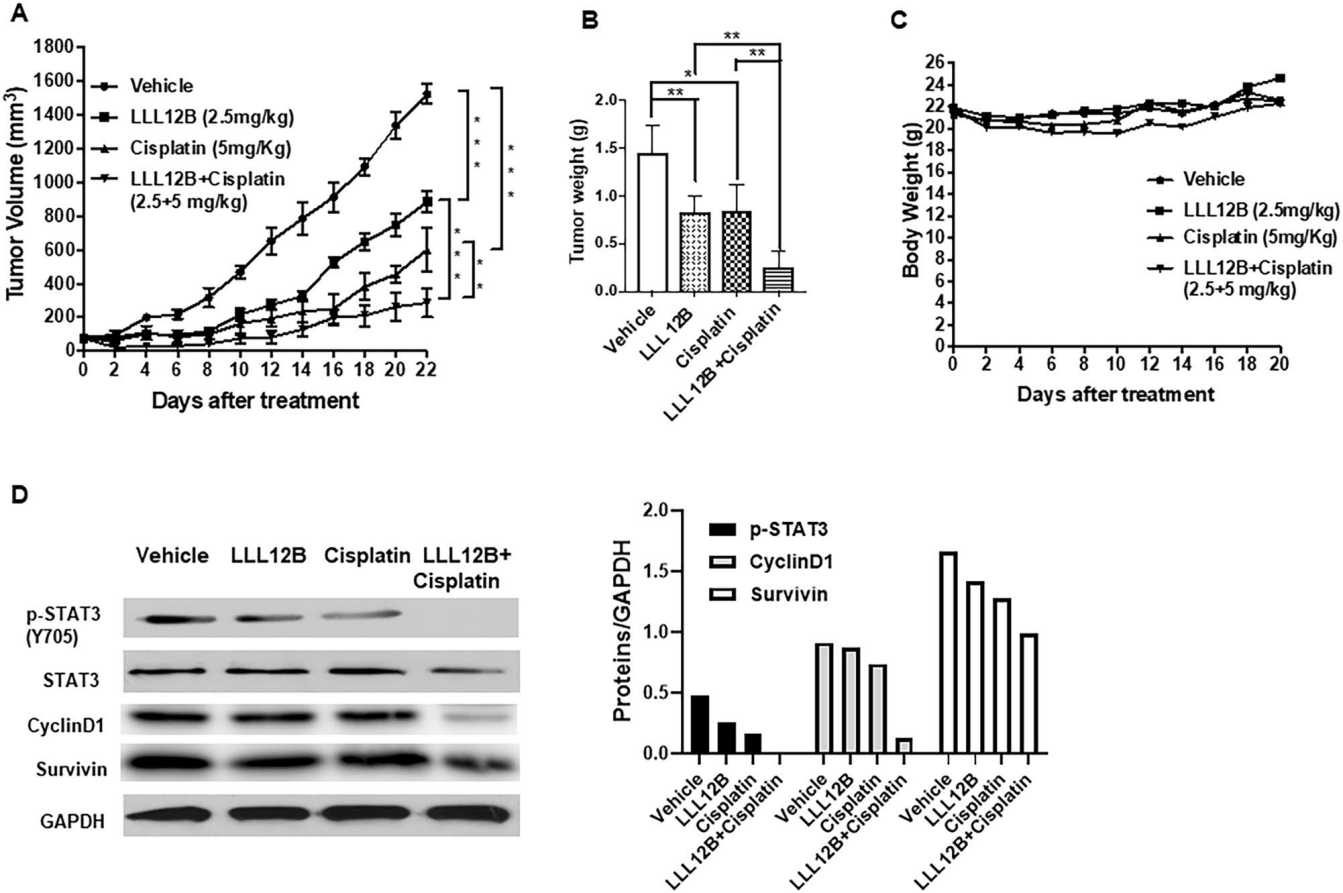
LLL12B and cisplatin combination suppressed tumor growth in D283 medulloblastoma xenografts. (**A**) Tumor volumes. (**B**) Tumor weights. (**C**) Body weight. (**D**) Western blots to analyze protein expression in the representative tumor samples. Expression levels of p-STAT3(Y705), STAT3, CyclinD1, Survivin, and GAPDH. D283 medulloblastoma cells (1 × 10^7^ cells/tumor) were implanted subcutaneously into the flank region of 4 to 5 week-old female athymic nude mice. Mice were randomized into four groups (5 mice for each group) and treated with LLL12B (2.5 mg/kg, oral gavage, once daily) and/or cisplatin (5 mg/kg, intraperitoneal injection, once every 4 days) or vehicle alone.

**Figure 10:**
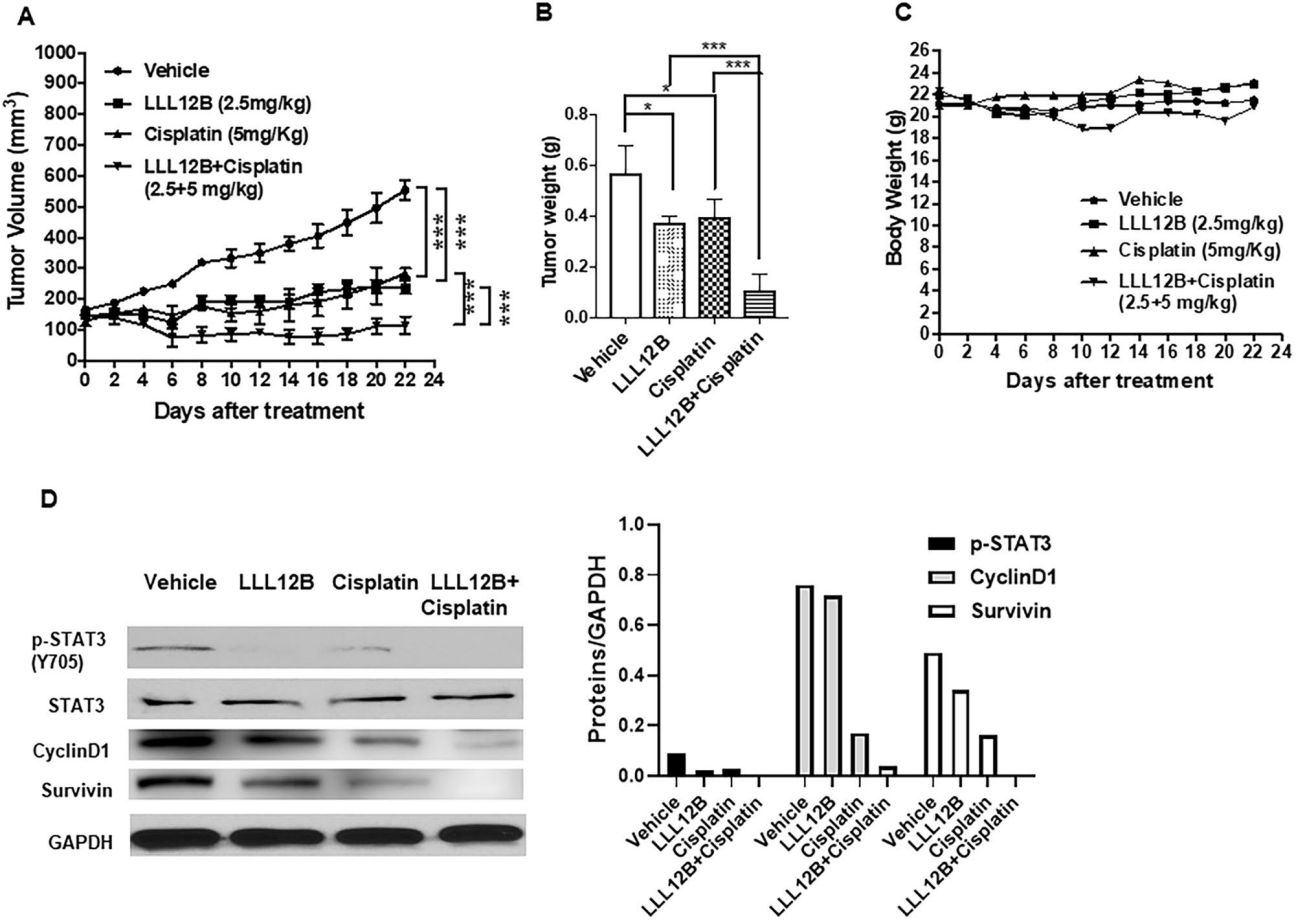
Anti-neoplastic activity of LLL12B and cisplatin combined treatment of D425 medulloblastoma xenografts. (**A**) Tumor volumes. (**B**) Tumor weights. (**C**) Body weights. (**D**) Protein analysis of the representative tumor samples by western blots. Expression levels of p-STAT3 (Y705), STAT3, CyclinD1, Survivin, and GAPDH. D245 medulloblastoma cells (1 × 10^7^ cells/tumor) were implanted subcutaneously into the flank region of 4 to 5 week-old female athymic nude mice. Mice were randomized into four groups (5 mice for each group) and treated with LLL12B (2.5 mg/kg, oral gavage, once daily) and/or cisplatin (5 mg/kg, intraperitoneal injection, once every 4 days) or vehicle alone.

To further verify the in vitro synergic anti-neoplastic activity of LLL12B in combination with cisplatin in vivo, nude mice tumor xenografts with subcutaneous implantation of D283 and D425 medulloblastoma cells were prepared. Mice were administrated with LLL12B (2.5 mg/kg) and cisplatin (5 mg/kg) alone or in combination. As shown in Figs. 9A and  10A, combination group dramatically inhibited tumor growth. After 22 days treatment, the tumor burden in combination groups were significantly decreased compared to the single drug groups in both D283 and D425 medulloblastoma xenograft models (Figs. 9B, 10B). Moreover, both the LLL12B and cisplatin single drug groups and the combination group have little effect on the body weights (Figs. 9C, 10C). Using western blots, it was confirmed that the expression level of STAT3 phosphorylation directly from tumors were reduced after LLL12B treatment. It was also showed that cisplatin alone could reduce or partially reduce the STAT3 phosphorylation^32^. Importantly, the drug combination further reduced the STAT3 phosphorylation levels (Figs. 9D, 10D). LLL12B and cisplatin combination also further reduce the expression of CyclinD1 and Survivin than single agent alone (Figs. 9D, 10D), which may provide a potential mechanism of drug combination is more effective in tumor growth suppressive activity than monotherapy (Figs. 9A, 10A).

## Discussion

STAT3 is a transcription factor and plays a critical role in tumorigenesis, which makes it as a potential molecular target in cancer therapy. Considerable effort has been made to develop effective inhibitors targeting STAT3 signaling. In our previous studies, we have developed and evaluated several small molecule STAT3 inhibitors, including LLL12, which blocks STAT3 phosphorylation and exhibits potent tumor growth-suppressive activity in cancer^24–27,29,33^. The report from Attarha et al. provided evidence to support that LLL12 binds to STAT3 protein in intact MCF-7 breast cancer cells^34^. So far, designing molecules that have good selectivity and bioavailability is still a difficult task and very few small molecule inhibitors have made their way to the clinic trials. In this study, we designed another STAT3 inhibitor named LLL12B using an AMLSD-based approach. LLL12B is a carbamate-based prodrug for LLL12. Our computational docking model of LLL12B and STAT3 also supported that LLL12B binding to STAT3 SH2 domain, which should be identical to that of LLL12. Importantly, LLL12B has one of the smallest molecular weights (374 dalton) compared to other STAT3 inhibitors. Furthermore, our study indicated that LLL12B exhibits superior in vivo PK and oral bioavailability than its parent STAT3 inhibitor LLL12. Blood–brain barrier (BBB) penetration prediction using CBLigand program predicted that LLL12B is able to penetrate BBB (SVM-MACCSFP BBB score is 0.062, which is greater than the minimum threshold of SVM-MACCSFP BBB score 0.020 that could pass through BBB)^35^. Moreover, our findings showed that LLL12B inhibits the STAT3 phosphorylation induced by IL-6 but does not inhibit the STAT1 phosphorylation stimulated by INF-γ. In addition, we demonstrated that the inhibition of P-STAT3 by LLL12B is very unlikely due to the only factor that LLL12B-treated cells reduce cell density by using same density of cells treated with LLL12B and treated for shorter time points (Fig. 3). LLL12B is very consistently inhibits P-STAT3 at 4 and 8 h time points and is not depend on cell density factor. The effects of LLL12B on P-AKT and P-ERK may be more cell line-dependent and the mechanism of these difference among cell lines are still needed for future investigation. Taken together, these results support that LLL12B is an excellent STAT3 inhibitor candidate for cancer therapy.

In this study, we tested LLL12B in human medulloblastoma cells, because medulloblastoma cells are sensitive to STAT3 inhibition. This is evidenced by the knock down of STAT3 by STAT3-specific siRNA can reduce cell viability in medulloblastoma cells. Interestingly, we found that STAT3 siRNA reduce P-STAT3 (Y705) expression level more than total STAT3 (Fig. 5A). This might be explained that there are a lot of more total STAT3 protein than phosphorylated STAT3 protein in the cells. STAT3 siRNA that reduce the abundant of total STAT3 protein, could significantly reduce STAT3 protein that are available to be phosphorylated at Tyr705. It is also possible that siRNA may reduce growth rate so that the cells were less confluent and secrete less IL6 to activate P-STAT3 (Y705). Both factors aforementioned may contribute to the dramatically reduction of P-STAT3 at Tyr705. Furthermore, because STAT3 activates its own promoter^36^, LLL12B that inhibits P-STAT3, could potentially reduce total STAT3. We next demonstrated that LLL12B exhibits anti-cancer cells activities through the inhibition of cancer cell viability and induction of apoptosis. Cell cycle gene CyclinD1 and survival gene Survivin are two downstream genes of STAT3 signaling pathway. Down-regulation of these two genes after LLL12B treatment could partially explain the mechanism of LLL12B-mediated inhibition cell viability through blocking the STAT3 signaling.

It has been reported that STAT3 signaling also have essential roles in cancer stem cells (CSCs)^13,37^, and medulloblastoma stem cells, which could grow as tumorsphere are considered to process the ability to sustain tumor growth^38,39^. In this study, we demonstrated that LLL12B inhibited tumorsphere formation in medulloblastoma cells, which may be related to a possible inhibitory effect on medulloblastoma stem cells^8,40^. Inhibiting STAT3 activity in the medulloblastoma stem cell population would be expected to inhibit tumor progression and the suppressive activity should be further enhanced when in combination with another anti-cancer agent.

Accordingly, combination of targeting of STAT3 pathway using small molecule inhibitors such as LLL12B and cytotoxic chemotherapy may provide improved therapy in medulloblastoma, because, chemo-resistance has been represented a major therapeutic challenge for recurrent medulloblastoma^41,42^. In addition, molecular and preclinical studies have indicated that the aberrant activation of STAT3 signaling pathways in recurrent tumors contributes to chemo-resistance in medulloblastoma^43^. Here, we sought to evaluate the effect of combining STAT3 inhibitor LLL12B and cisplatin, which is a platinum-based chemotherapeutic agent used in frontline treatment regimens for brain tumors of childhood including medulloblastoma^5^. Our results indicated that cisplatin reduced STAT3 phosphorylation. Turkson et al., suggested that platinum compounds could disrupt STAT3 DNA-binding activity in cells, but the exact mechanism by which cisplatin reduces P-STAT3 still needs further investigation^32^. Our results demonstrated that this combination exhibited synergistic activity in the inhibition of cell viability in all 4 medulloblastoma cell lines tested (CI < 1.0).

Apoptosis is associated with the overall integrity of multicellular organisms, and when altered it can contribute to tumorigenesis. Inactivation of proapoptostic proteins or increased expression of anti-apoptotic proteins is considered as the leading cause of chemoresistance encountered during cancer therapy^44^. There are two types of pathways that can trigger apoptosis, the extrinsic pathway and intrinsic pathway, which operate via a variety of death receptors and apoptogenic factors^45–47^. Some caspases also play crucial roles in the activation and execution of programmed cell death, especially caspase-3 and caspase-7. Indeed, caspase-3 and caspase-7 are the main executioners in apoptotic cells as they can be activated through both extrinsic and intrinsic signaling pathways and might be more important in most of the downstream events of the apoptotic pathways^37,48^. Therefore, the activation of caspase-3/7 is considered as a key marker of apoptosis. Our present data showed that active caspase-3/7 was increased after LLL12B treatment in medulloblastoma cells, suggesting the induction of apoptosis by LLL12B. In addition, LLL12B and cisplatin combination demonstrated greater induction of apoptosis in medulloblastoma cells in vitro and greater suppression of tumor growth in medulloblastoma xenografts in vivo. In a pilot orthotopic tumor model in vivo using D425 medulloblastoma cells, we observed that LLL12B showed limited extension of the survival of mice with tumors. The survival in LLL12 treated mice were averaged for 24 days versus 19 days of vehicle treated mice. In the future, it will be interested and helpful to extend this study of testing LLL12B and cisplatin combination in orthotopic medulloblastoma model in vivo.

In summary, we have developed a novel small molecule STAT3 inhibitor, LLL12B, which is orally bioavailable in vivo. Our results demonstrated that LLL12B has potential therapeutic benefit in the treatments of medulloblastoma. As a single agent, LLL12B showed an effective inhibition in tumor growth and anti-apoptotic effects through the inhibition of STAT3 signaling in vitro and in vivo in medulloblastoma cells. We also provided evidence that LLL12B exhibited selectivity toward STAT3 but not to STAT1. These results support LLL12B is a potential lead candidate for further development and evaluation. Furthermore, our data showed that LLL12B and cisplatin combination exhibit greater inhibition of cell viability and tumorsphere growth, induction of apoptosis of medulloblastoma cells in vitro and suppression of medulloblastoma tumor growth in vivo. Overall, our data provided experimental evidence indicating novel STAT3 inhibitor LLL12B in combination with cisplatin is a potential therapeutic approach for medulloblastoma in the future.

## Supplementary Information

Supplementary Information

## Data Availability

The datasets generated during and/or analyzed during the current study are available by request.
