# Supplementary material for: LLL12B, a small molecule STAT3 inhibitor, induces growth arrest, apoptosis, and enhances cisplatin-mediated cytotoxicity in medulloblastoma cells
Source: Sci Rep. 2021 Mar 22;11:6517. doi: 10.1038/s41598-021-85888-x (PMC7985203; doi:10.1038/s41598-021-85888-x)

Supplementary Information

**Figure 2 Original western blot images**

D283-p-STAT3


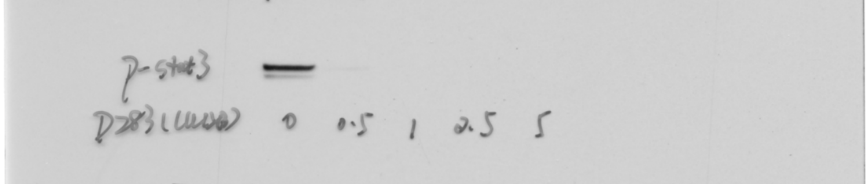


D283 STAT3 and GAPDH


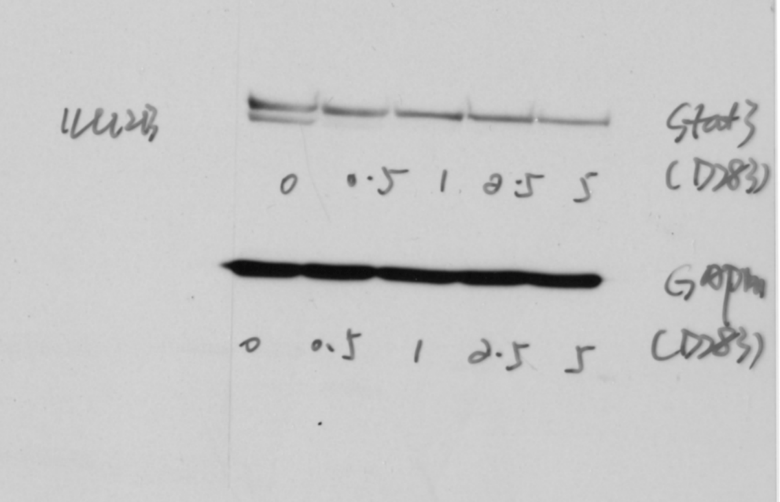


D283-Survivin


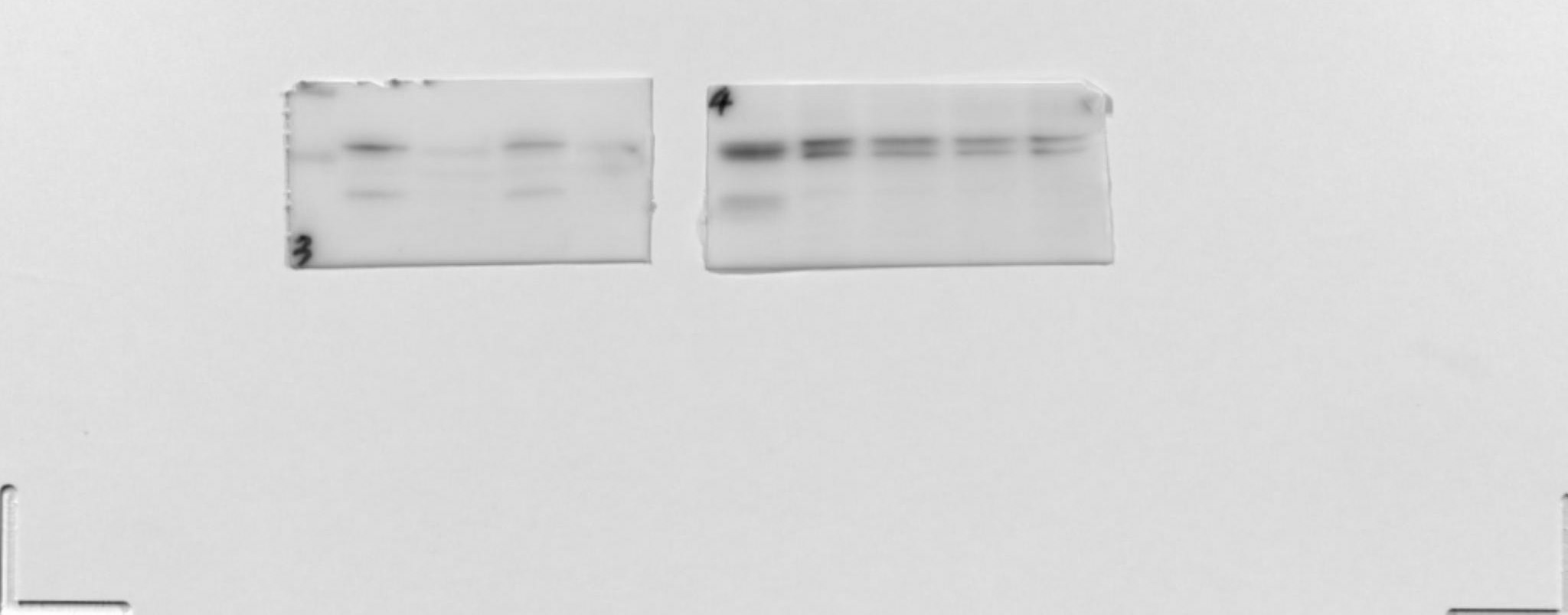


D283-CyclinD1


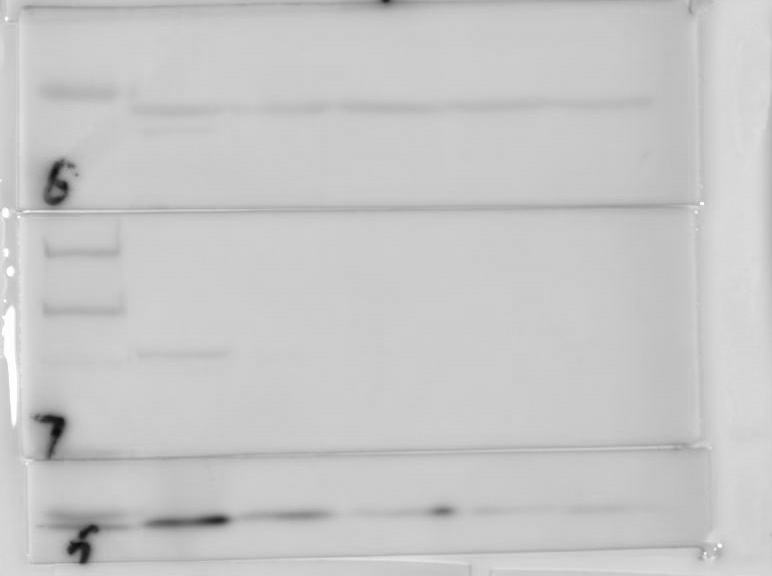


D425-P-STAT3


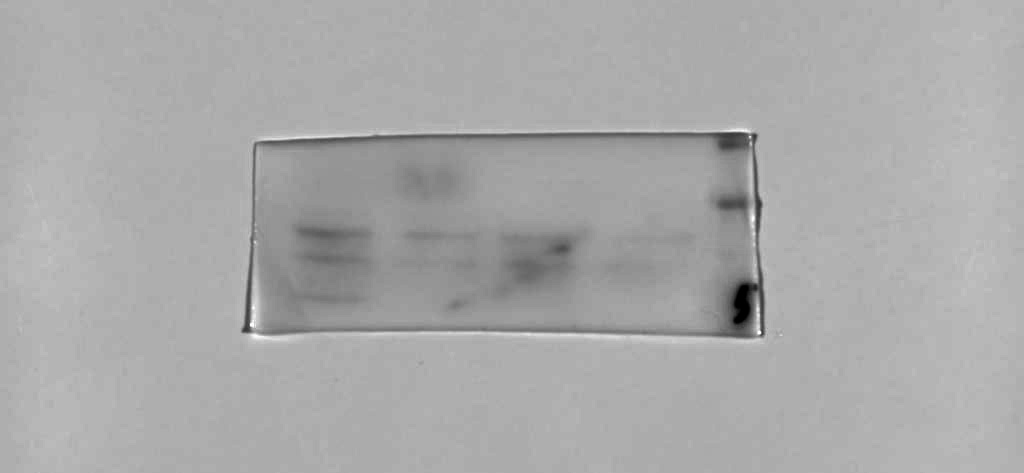


D425-STAT3


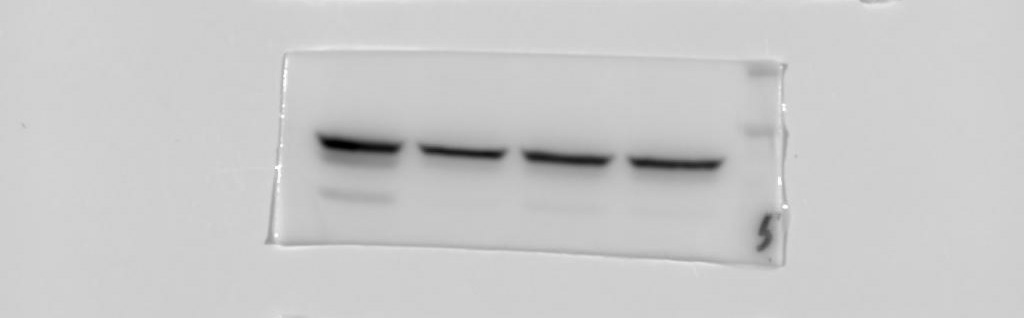


D425-Survivin


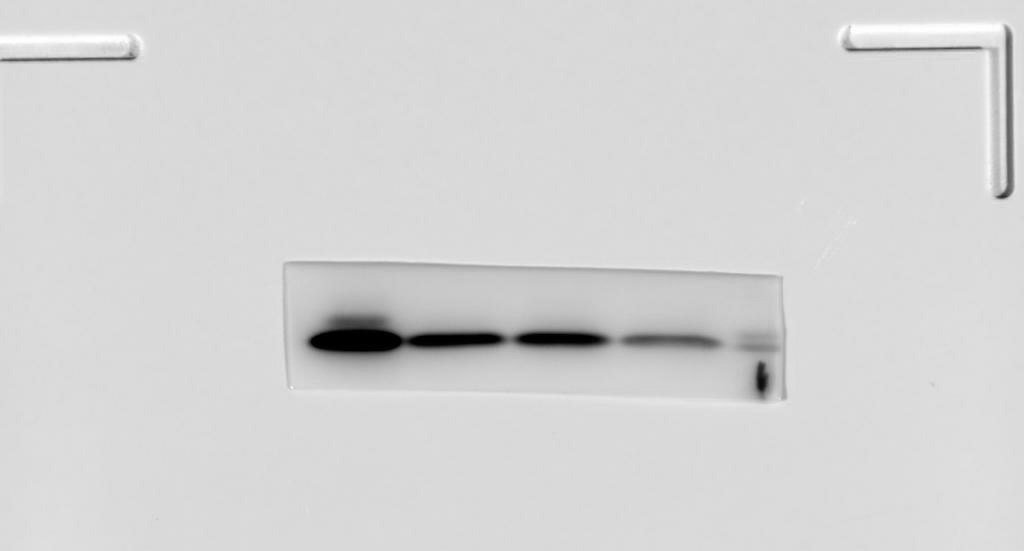


D425-CyclinD1


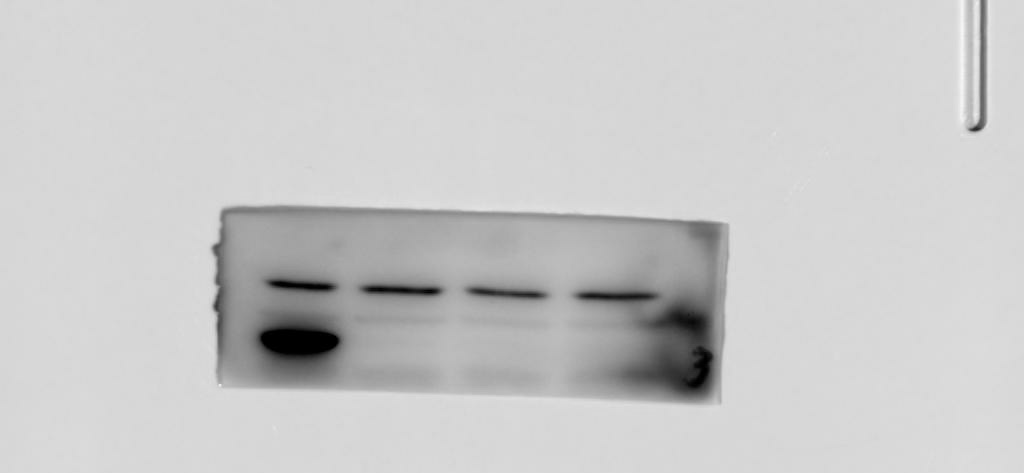


**Figure 3 Original western blot images**

UW426-P-STAT3


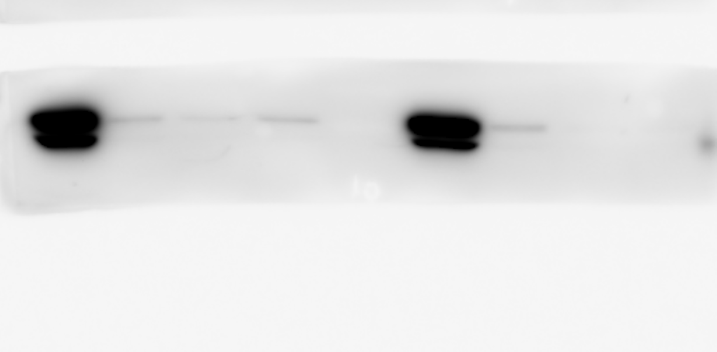


UW426-STAT3


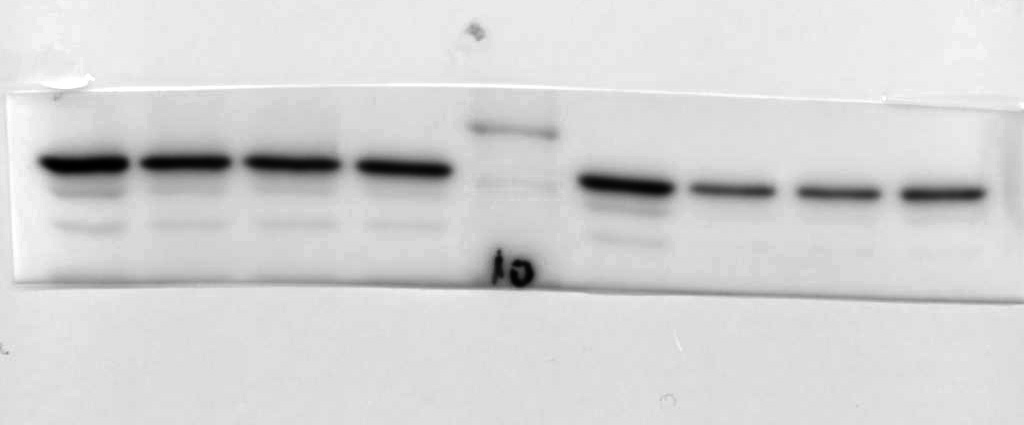


UW426 P-ERK


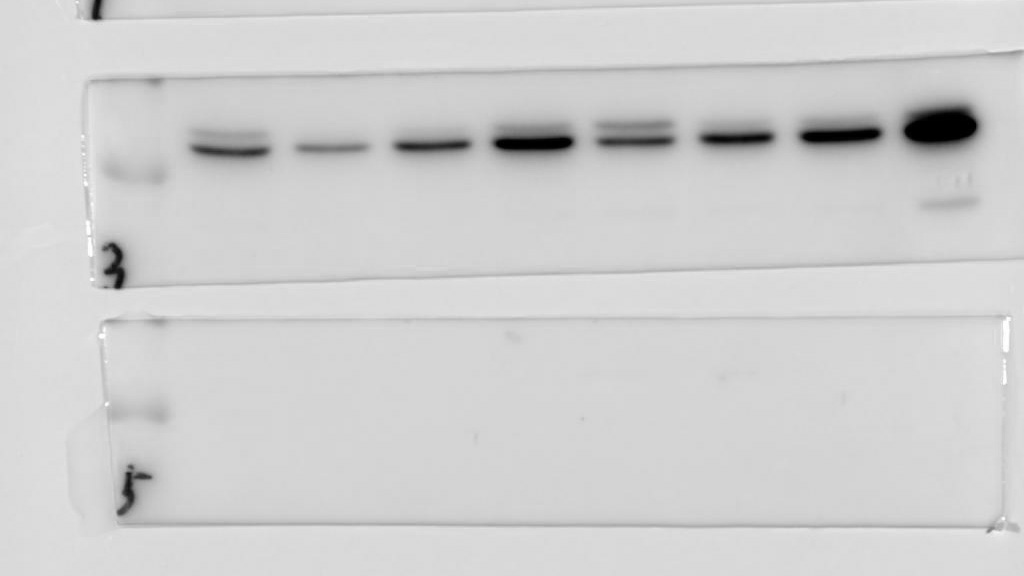


UW426 GAPDH


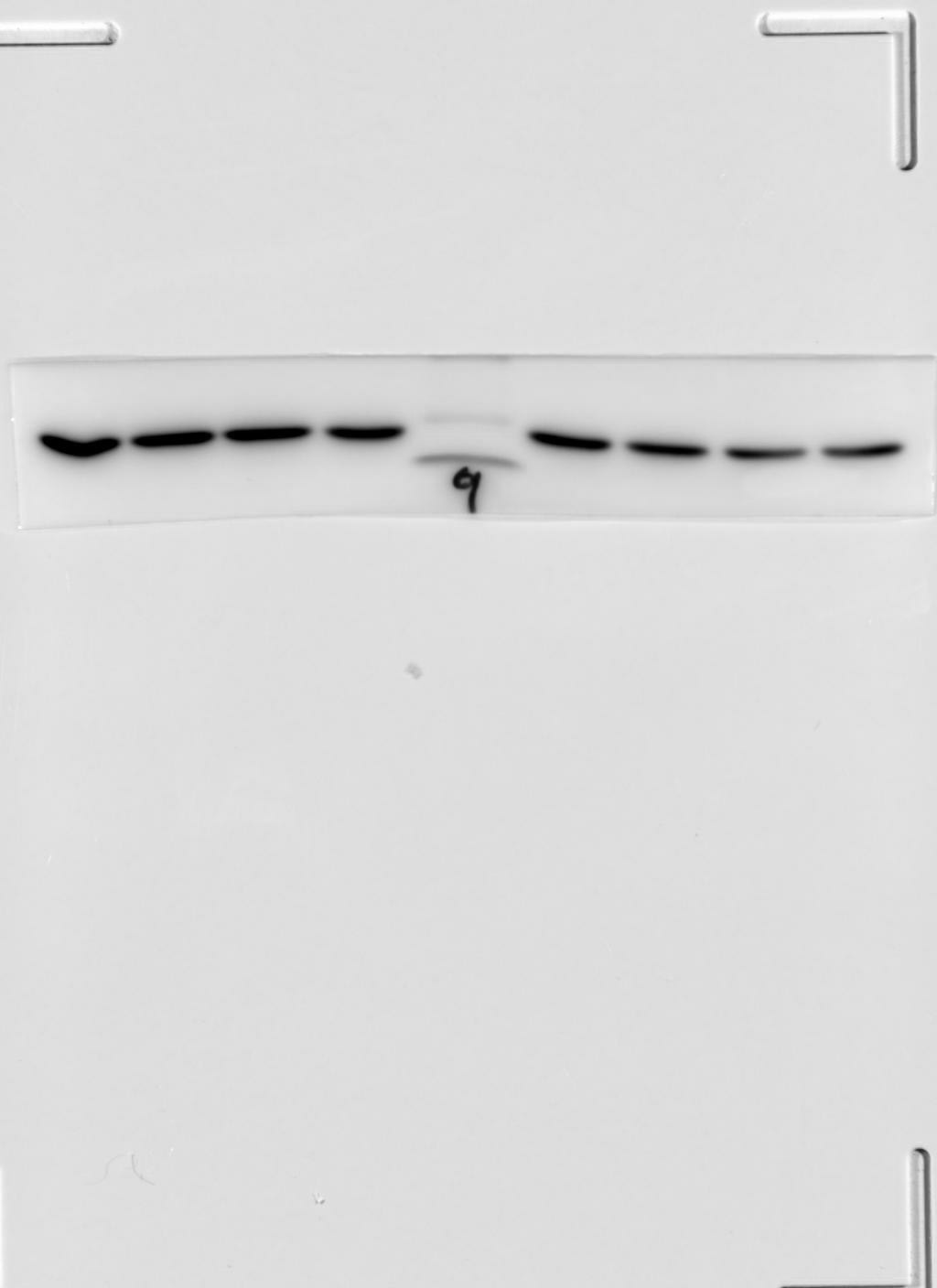


UW288 and D283 P-STAT3


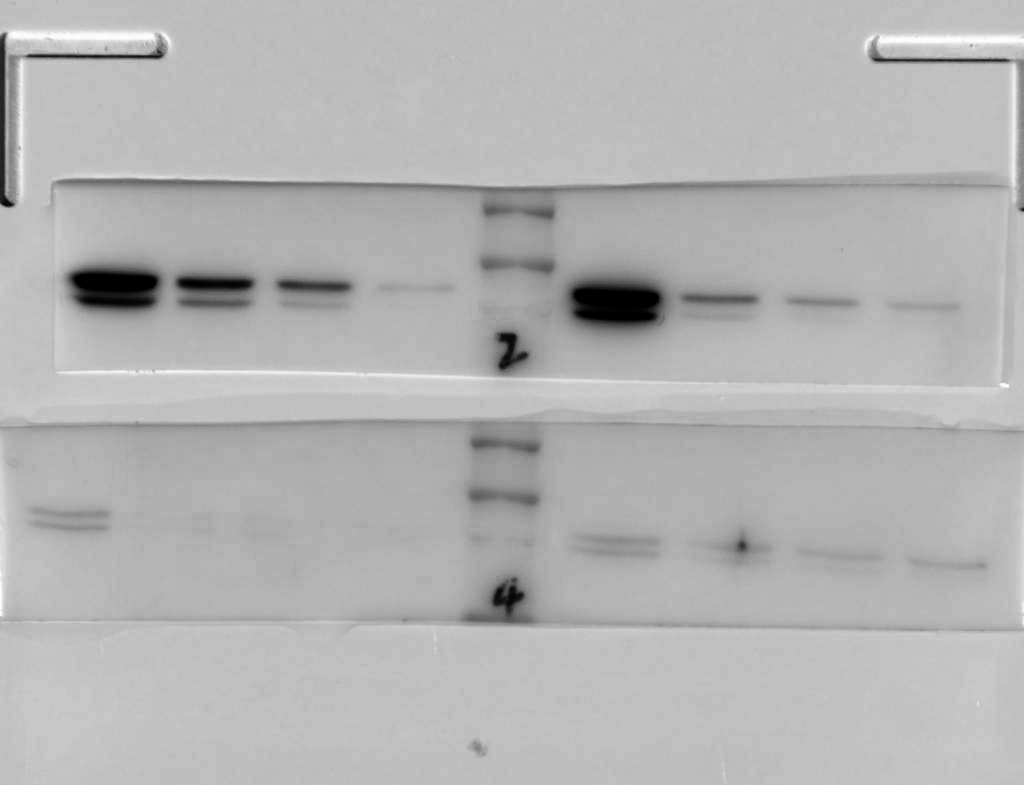


UW288 and D283 STAT3


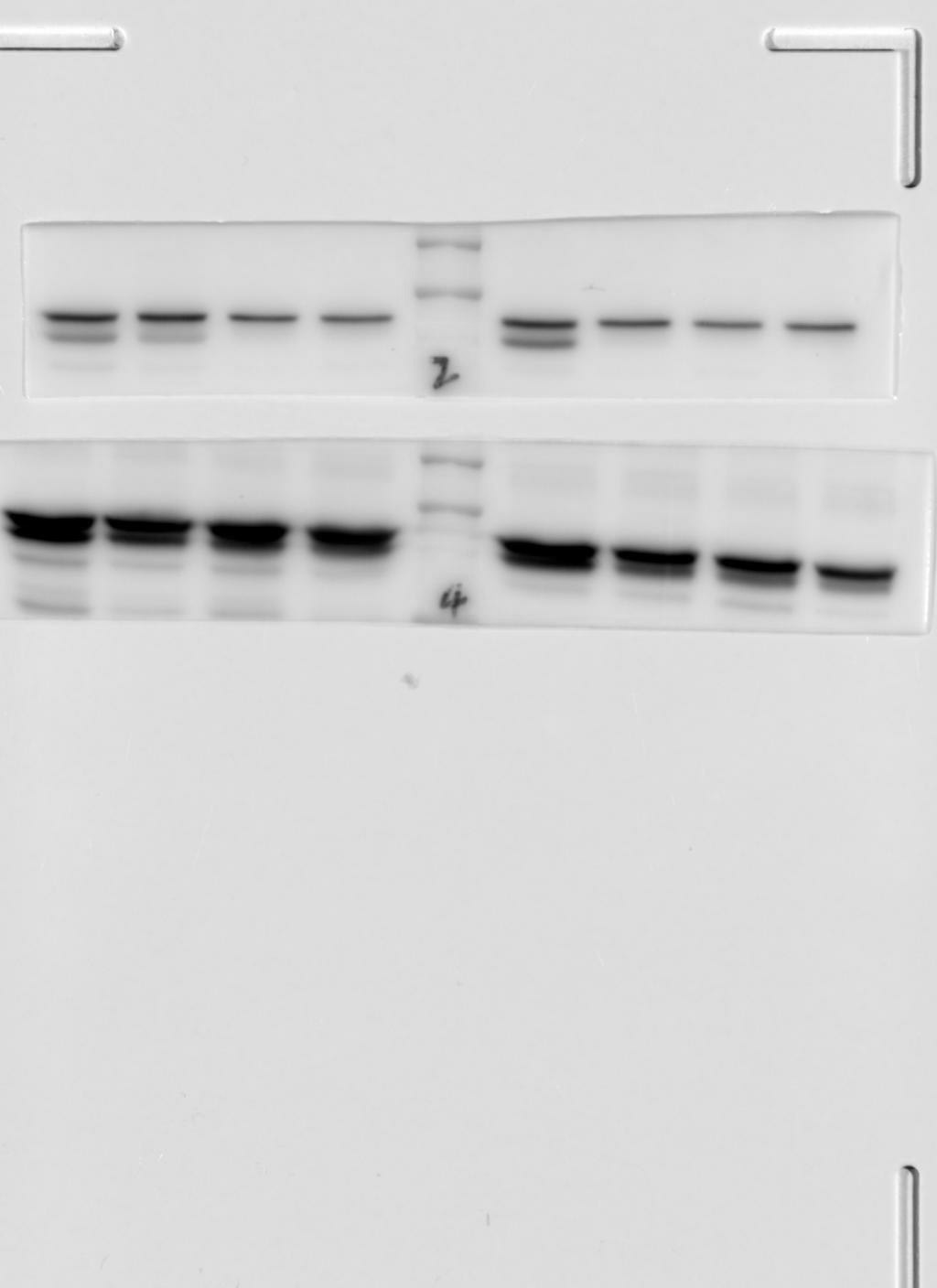


UW288 P-ERK


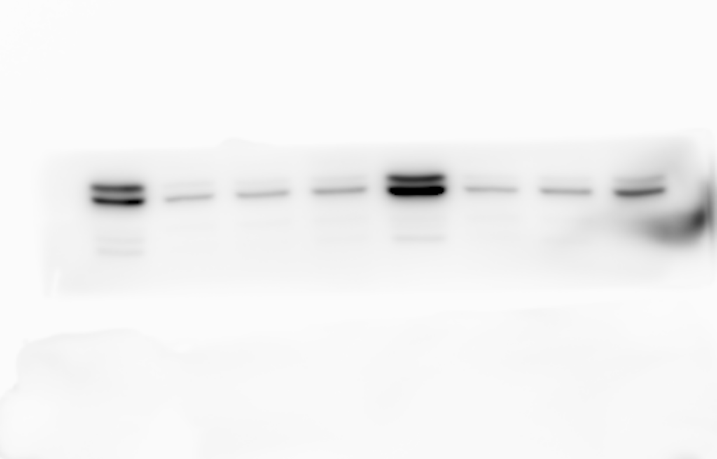


UW288 and D283 GAPDH


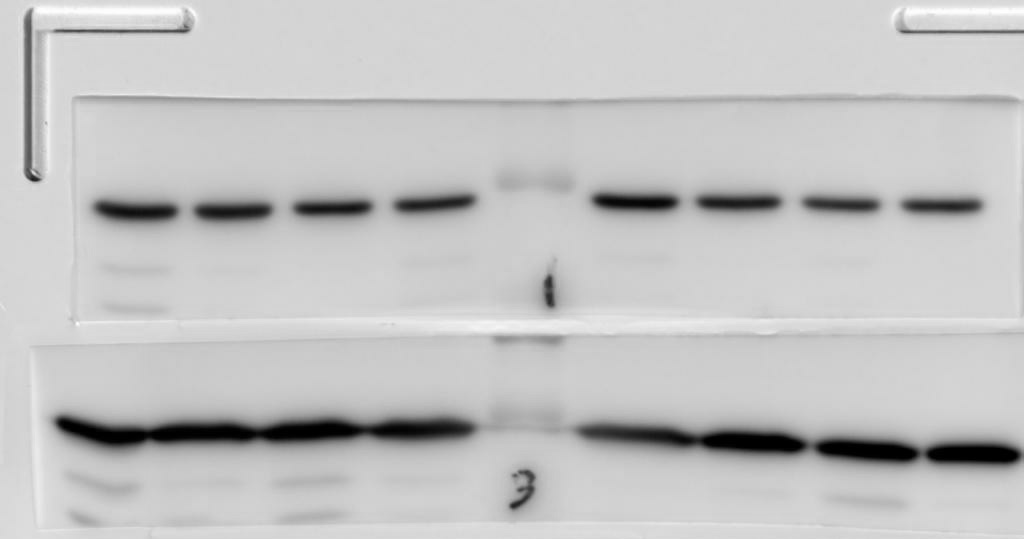


D283 P-ERK


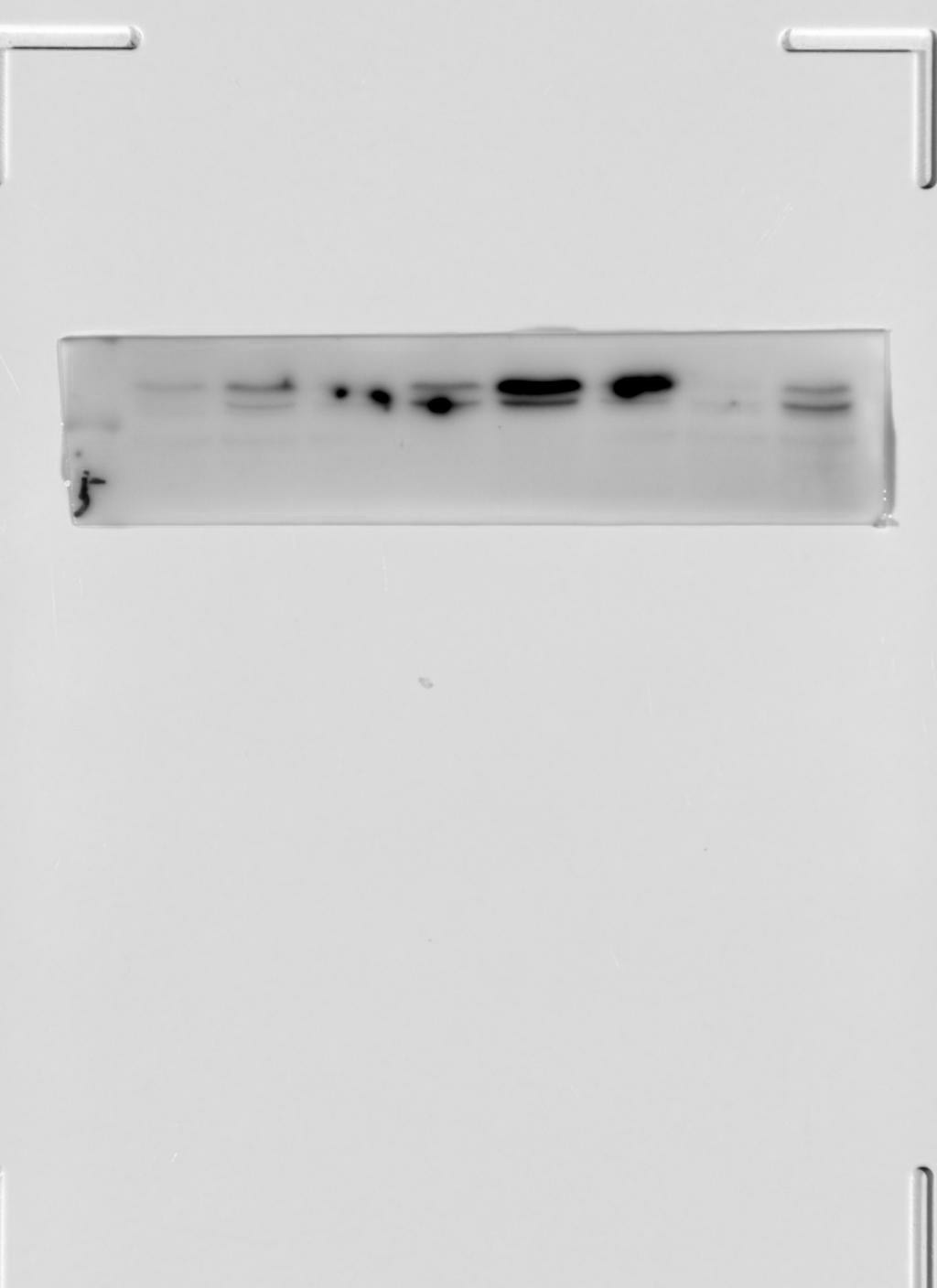


UW426, UW288 and D283 ERK


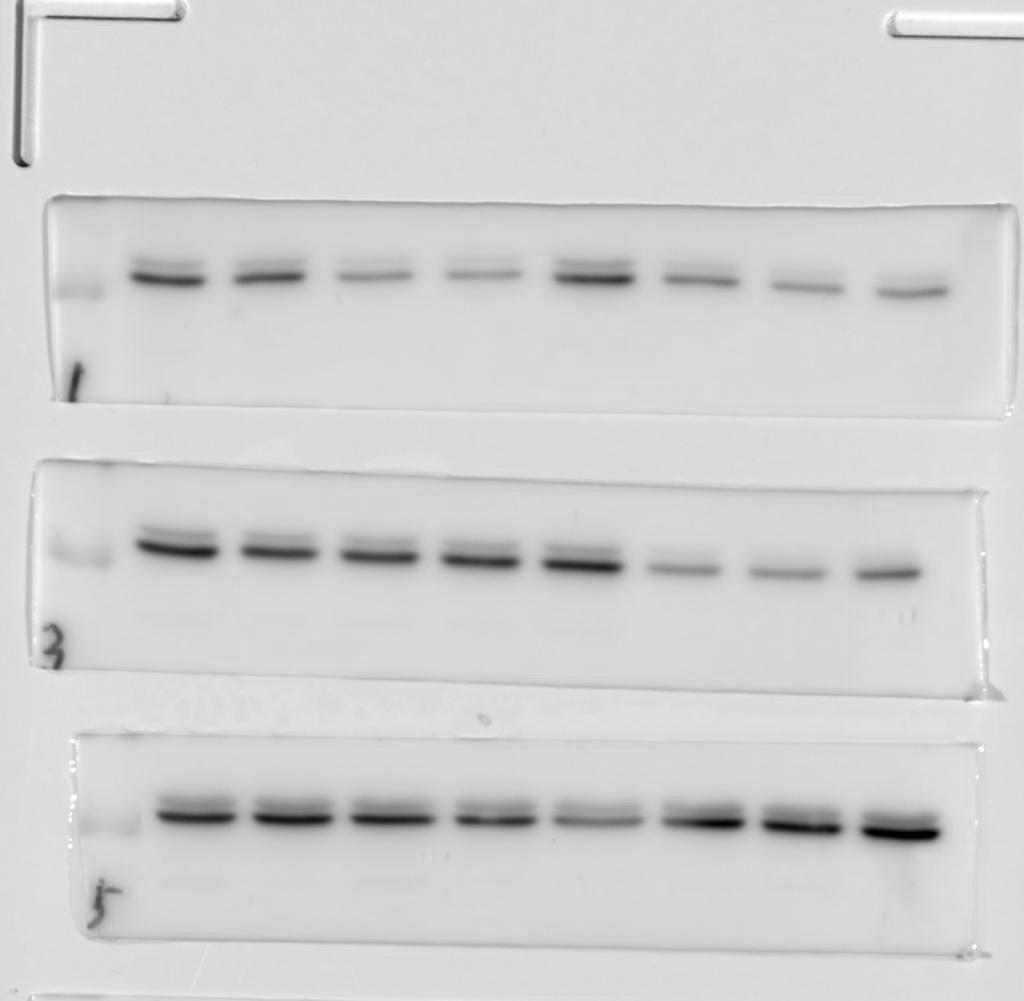


UW426, UW288 and D283 P-AKT


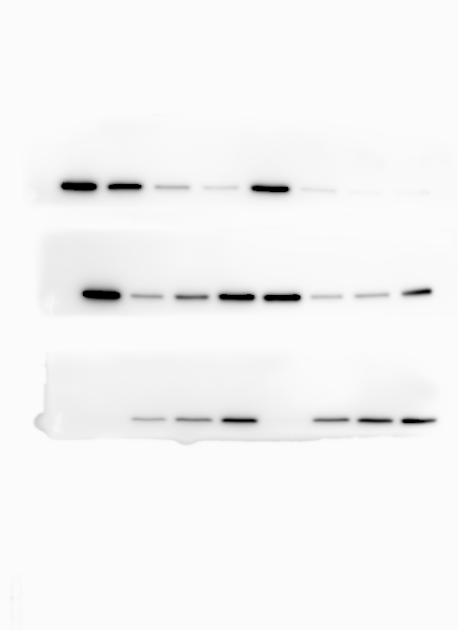


UW426, UW288 and D283 AKT


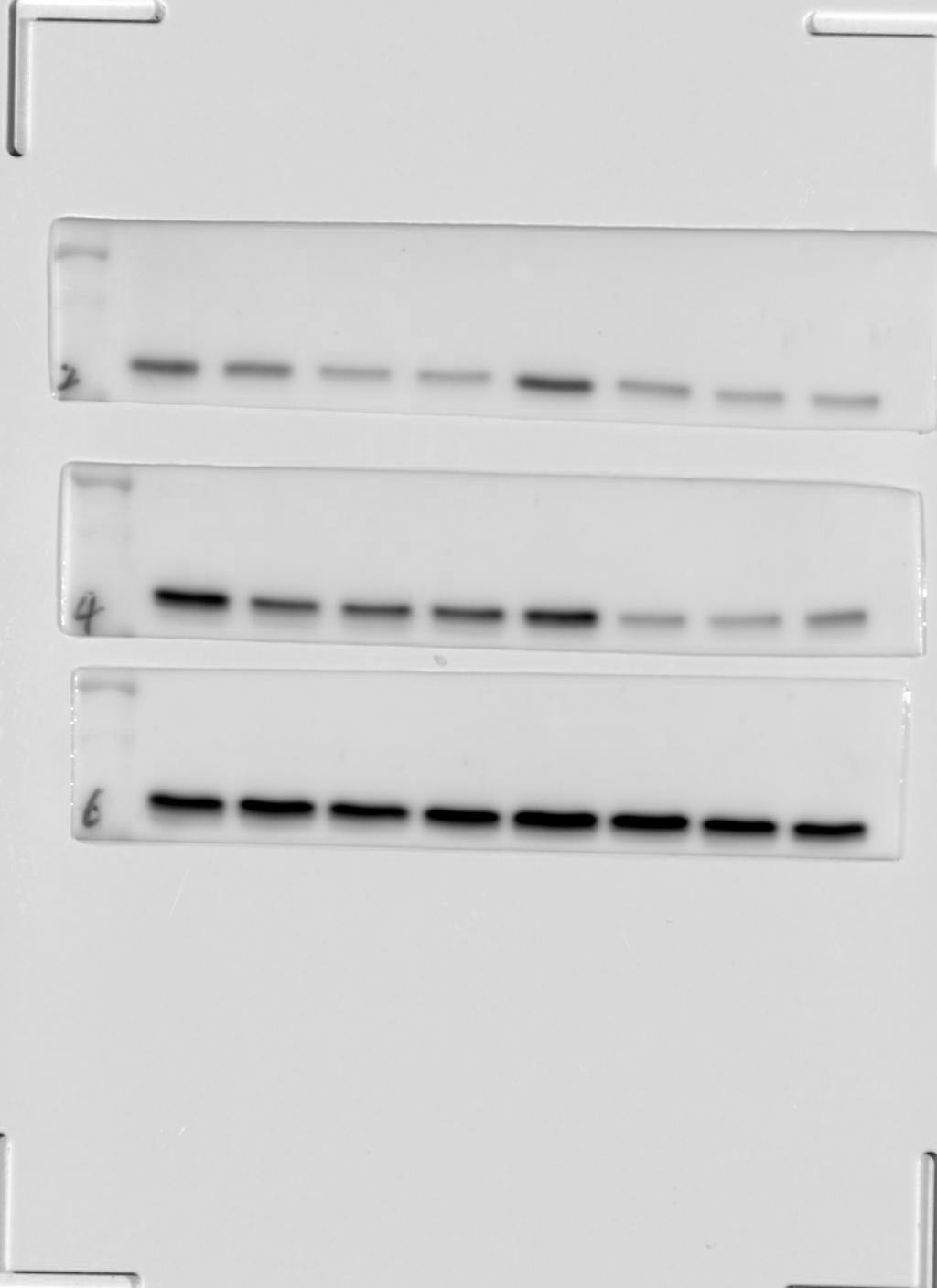


**Figure 5 Original western blot images**

D283 P-STAT3 the last two lanes


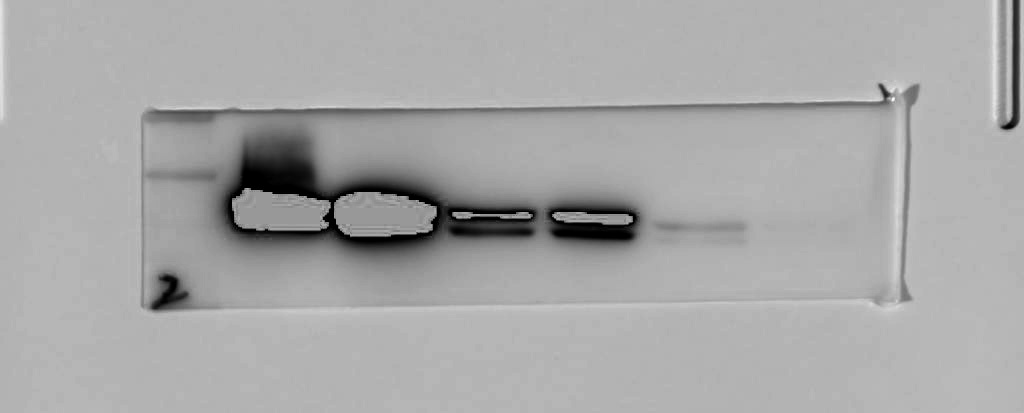


D283 STAT3 the last two lanes


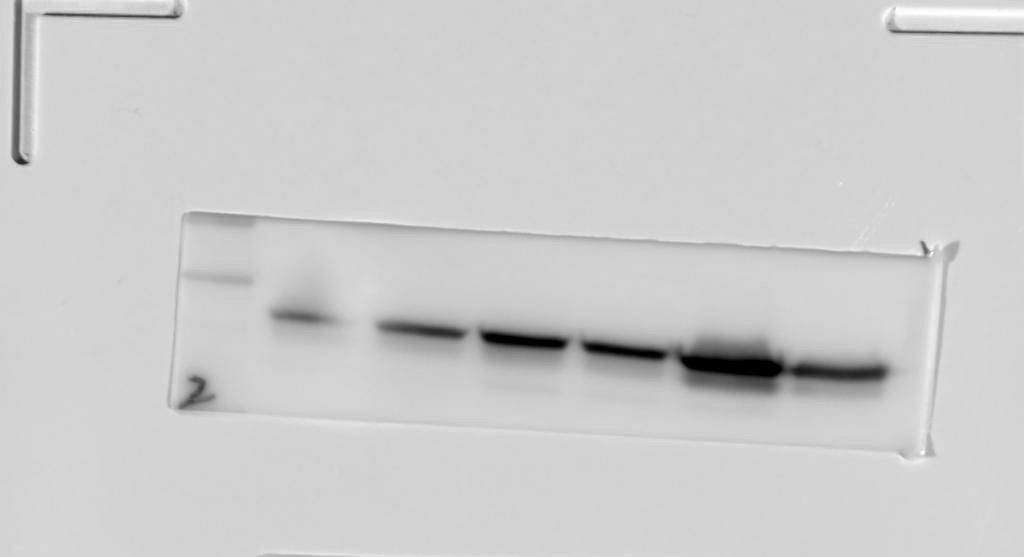


D283 GAPDH the last two lanes


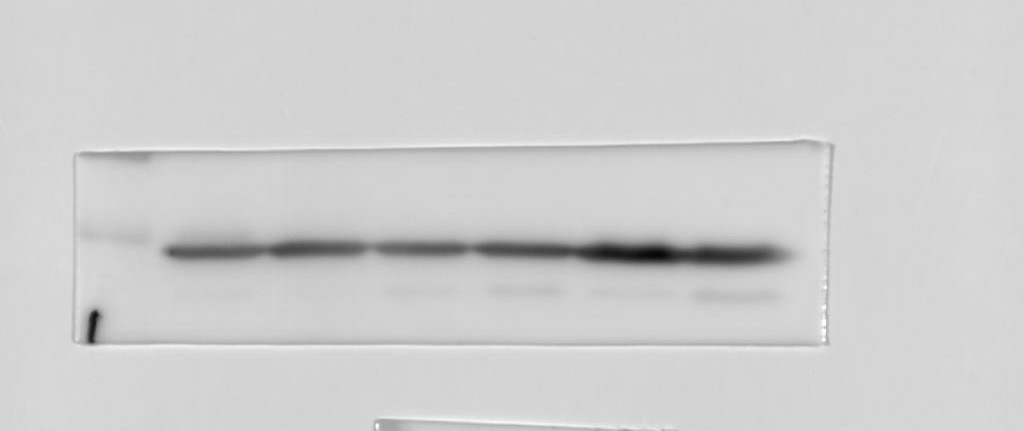


D425 P-STAT3 the first two lanes


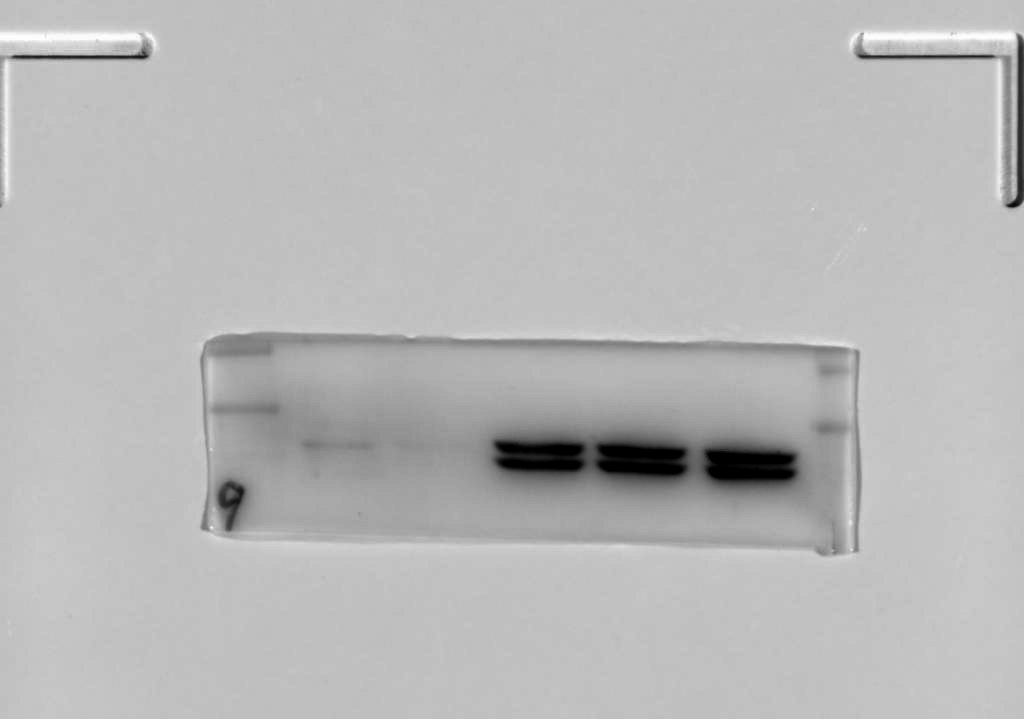


D425 STAT3 the first two lanes


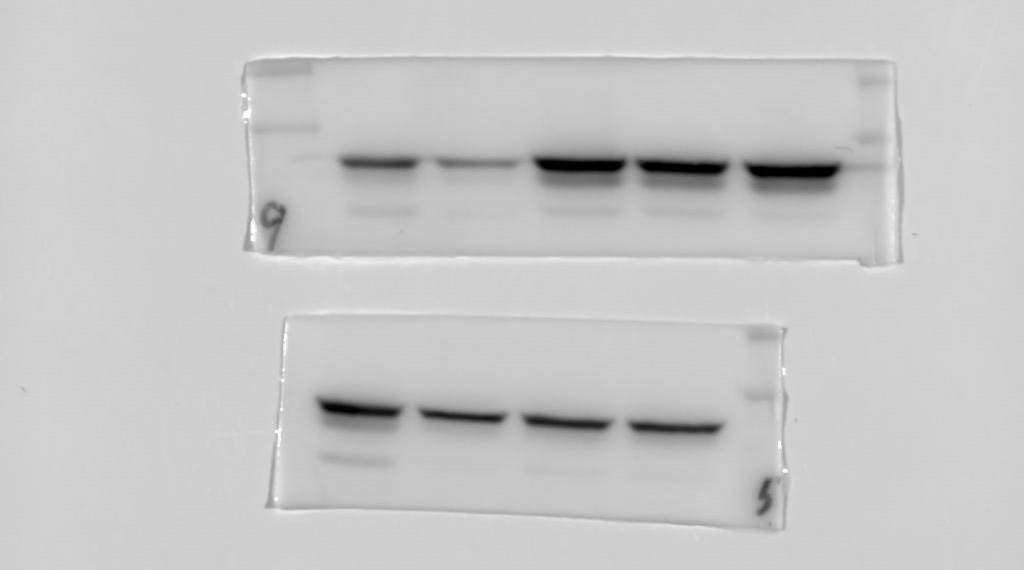


D425 STAT3 the first two lanes


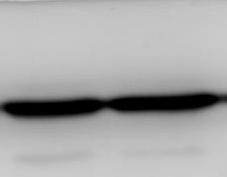


UW288 P-STAT3 the last two lanes


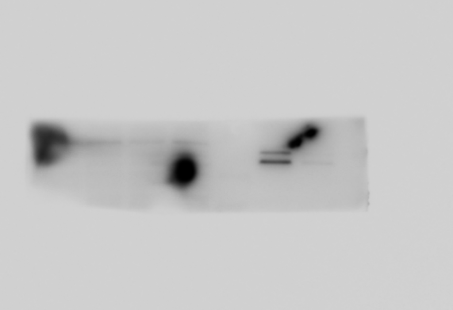


UW288 STAT3 the last two lanes


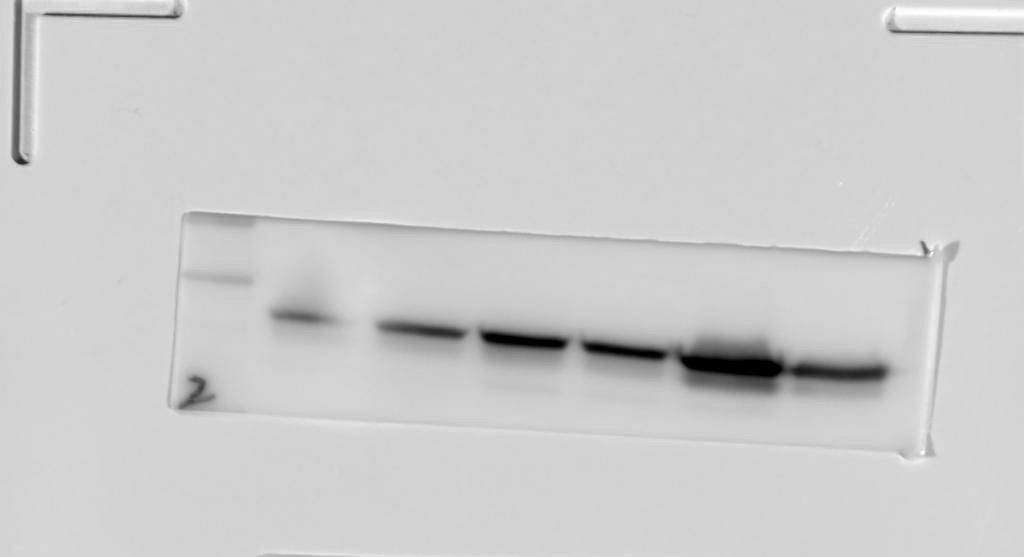


UW288 GAPDH the last two lanes


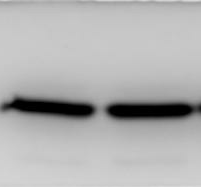

Supplement: Supplementary file 1 — Supplementary Information [file 41598_2021_85888_MOESM1_ESM.docx]
